# Fatty Acid Concentration and Phase Transitions Modulate A*β* Aggregation Pathways

**DOI:** 10.1038/s41598-017-09794-x

**Published:** 2017-09-04

**Authors:** Pratip Rana, Dexter N. Dean, Edward D. Steen, Ashwin Vaidya, Vijayaraghavan Rangachari, Preetam Ghosh

**Affiliations:** 1 Department of Computer Science, Virginia Commonwealth University, Richmond, VA 23284 USA; 2 Department of Chemistry & Biochemistry, University of Southern Mississippi, Hattiesburg, MS 39406 USA; 3 Department of Mathematical Science, Montclair State University, Montclair, NJ 07043 USA

## Abstract

Aggregation of amyloid *β* (A*β*) peptides is a significant event that underpins Alzheimer disease (AD) pathology. A*β* aggregates, especially the low-molecular weight oligomers, are the primary toxic agents in AD and hence, there is increasing interest in understanding their formation and behavior. Aggregation is a nucleation-dependent process in which the pre-nucleation events are dominated by A*β* homotypic interactions. Dynamic flux and stochasticity during pre-nucleation renders the reactions susceptible to perturbations by other molecules. In this context, we investigate the heterotypic interactions between A*β* and fatty acids (*FA*s) by two independent tool-sets such as reduced order modelling (ROM) and ensemble kinetic simulation (EKS). We observe that *FA*s influence A*β* dynamics distinctively in three broadly-defined *FA* concentration regimes containing non-micellar, pseudo-micellar or micellar phases. While the non-micellar phase promotes on-pathway fibrils, pseudo-micellar and micellar phases promote predominantly off-pathway oligomers, albeit via subtly different mechanisms. Importantly off-pathway oligomers saturate within a limited molecular size, and likely with a different overall conformation than those formed along the on-pathway, suggesting the generation of distinct *conformeric strains* of A*β*, which may have profound phenotypic outcomes. Our results validate previous experimental observations and provide insights into potential influence of biological interfaces in modulating A*β* aggregation pathways.

## Introduction

Aggregation of A*β* constitutes the central process in Alzheimer disease (AD) pathology. Brains of AD patients contain large amounts of proteinacious plaques mainly comprised of insoluble A*β* fibril deposits. Monomeric A*β* peptides (A*β*40 and A*β*42) spontaneously undergo aggregation towards large fibrils in a nucleation dependent manner. The effects of nucleation on aggregation dynamics have been extensively studied^1–8^ that point to a key rate-limiting step for the formation of nucleus/nuclei^9–13^. Therefore, the dynamics associated with reactions leading up to nucleation is critical for aggregation. Energetically, the pre-nucleation phase is nebulous with monomers and oligomers (dimers, trimers etc.) involved in a dynamic flux dominated by stochastic interactions^14–16^. Furthermore, intrinsic disorder and amphipathic nature of A*β* facilitate multiple phase transitions and heterogeneous interactions during nucleation, making the process particularly sensitive to environmental factors such as pH, ionic strength, temperature and other interacting partners^17–22^. This is significant because smaller, soluble aggregates have emerged as the primary neurotoxic agents responsible for memory loss in AD^23–26^. Furthermore, it is clear that oligomers may not be the obligatory intermediates to fibril formation, and that the oligomers can also be populated along alternate pathways of aggregation (off-pathways)^22, 27–32^. Off-pathway oligomers differ from those formed along the on-pathway resulting in multiple conformational variants with distinct biochemical and cellular properties. Given the conformational diversity among oligomers, it is imperative to determine the factors that affect dynamics involved in such oligomer formation to establish a framework of molecular mechanisms that better defines amyloid progression.

**Figure 1:**
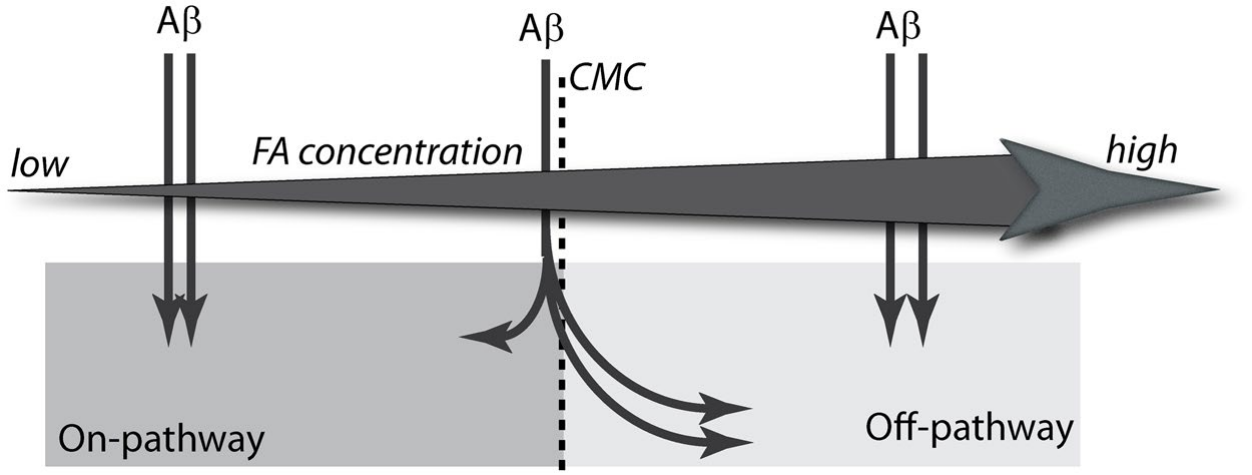
Schematic of the switching dynamics between the on- and off-pathways.

One class of biologically important interacting partners that affect A*β* pre-nucleation dynamics are the anionic surfactants such as fatty acids and lipids^33–39^. Lipids and fatty acids are profoundly important in physiological contexts as they are abundant in both brain tissues and CSF^40, 41^. The amphipathic A*β* peptide is known to have strong affinity for membranes and hence, these interactions may affect the pre-nucleation dynamics. Several reports also demonstrate the effects of phospholipids on A*β* aggregation^42–44^. Similarly, both polyunsaturated (PUFAs) and saturated fatty acids are also known to have significant effects on the AD brain^45, 46^. Kumar and co-workers have previously reported the generation of 4–5 mers and 12–24 mers from lauric acid along a non-fibril formation pathway^22^. More importantly, using carbon chain lengths of C9-C12 fatty acids (*FA*s), they established that below (non-micellar), near (pseudo-micellar) and above (micellar) their respective critical micelle concentrations (CMCs), *FA*s generate A*β* oligomers or fibrils via distinct pathways^22^. A schematic of the switching dynamics from on- to the off-pathway with increasing concentration of *FA*s is shown in Fig. 1. Here the bold horizontal arrow signifies increasing *FA* concentration while the vertical arrows conceptually depict the dynamical aggregation regimes of the system with the inclusion of monomeric *Aβ*. It can be observed that low *FA* concentration involves the non-micellar phase where only the on-pathway reactions are active; while high *FA* concentration (beyond the CMC) involves the micellar phase where only the off-pathway reactions are active (with on-pathway being completely switched off). The middle phase of the *FA* concentration arrow denotes the near-CMC (i.e., pseudo-micellar) phase and is the most interesting one; here, both on- and off-pathway reactions are active and the monomeric *Aβ* may form aggregated oligomers from both pathways simultaneously (as denoted by the arrows).

In this report, we sought to understand the dynamics of heterotypic interactions between A*β* and *FA*s and how phase transitions of *FA*s modulate A*β* aggregation. We first use a reduced order model (ROM) and a corresponding mathematical analysis rooted in a linear stability argument to identify the switching behavior from on- to off-pathway in the presence of micellar interfaces. The ROM can be thought of as a toy-version of the far more complex biophysical problem. However, despite considering only 4-species in this model, the ROM still deals with a highly non-linear problem which captures the overall aggregation dynamics of the Aβ system and allows for rigorous examination of features such as dynamic stability which would be intractable in the case of a very fine grained model. Mathematical biologists consistently simplify complex biological problems to gain initial insight into problems, to understand the nature of equilibria, stability, bifurcations in the larger system, before venturing to speak of specific details which is the objective of the ROM. This analysis was used as the basis to further develop a more detailed model, which was built around the discoveries of the ROM and experimental data.

Secondly, using an ensemble kinetic simulation (EKS) model, we demonstrate that in three distinct phase-transition regimes, *FA*s significantly and consistently modulate A*β* aggregation by altering the pathways. These results are in agreement with the reported *in vitro* experimental observations, and provides detailed molecular insights into the heterotypic interactions between A*β* and *FA*s. These insights also reveal that generation of conformationally-distinct oligomeric strains (produced by the oligomers involved in the on- and off-pathways) are highly likely in physiological environments containing lipid and *FA* interfaces due to the dynamics involved in the heterotypic *FA*-A*β* interactions.

## Results

### Stability analysis for a reduced order model (ROM)

We examined the sets of on- and off-pathway reactions shown below. The model system considered is as follows:

1 $${A}_{1}+FA\mathop{\mathop{\rightleftharpoons }\limits_{{k}_{1}^{-}}}\limits^{{k}_{1}^{+}}{A}_{1}^{^{\prime} }$$

2 $$n\cdot {A}_{1}\mathop{\mathop{\rightleftharpoons }\limits_{{k}_{2}^{-}}}\limits^{{k}_{2}^{+}}{A}_{n}$$

3 $$n\cdot {A}_{1}^{^{\prime} }\mathop{\mathop{\rightleftharpoons }\limits_{{k}_{3}^{-}}}\limits^{{k}_{3}^{+}}{A}_{n}^{^{\prime} }$$

where the reaction 2 is referred to the on-pathway, while reaction 3 is the off-pathway. In these reactions, *A*_1_ denotes the A*β* monomers while ${A}_{1}^{^{\prime} }$ denotes the off-pathway monomers created in the presence of fatty acids, *FA* (considering critical micelle concentration, CMC). *A*_*n*_ are the on-pathway oligomers while ${A}_{n}^{^{\prime} }$ are the off-pathway oligomers made of *n* monomeric units. Finally, ${k}_{i}^{+}$ and ${k}_{i}^{-}$ are the forward and backward rate constants of the corresponding reactions (*i* = 1, 2, 3). The details of the modeling methodology are elaborated in the Methods section. We are aware that the on-pathway fibrils are considerably larger than the terminal species of the off-pathway, i.e. size of (*A*_*n*_) ≫ size of (*A* ^′^_*n*_) or *n* ≫ *n*′. In the ROM analysis however, both pathways terminate at the same value of *n* since we believe this to be sufficient to elucidate the competition between the two pathways in our model. Since the only “bridge” from the on- to off-pathway is between the monomeric species, once the reaction chooses the path from ${A}_{1}\to {A}_{n}$, the on-pathway dominates in the steady state (or vice-versa) and higher order oligomer dynamics do not reveal anything more about the process. The asymmetric nature of the two pathways is however, considered in our follow-up analysis to understand detailed mechanistic phenomena.

**Figure 2:**
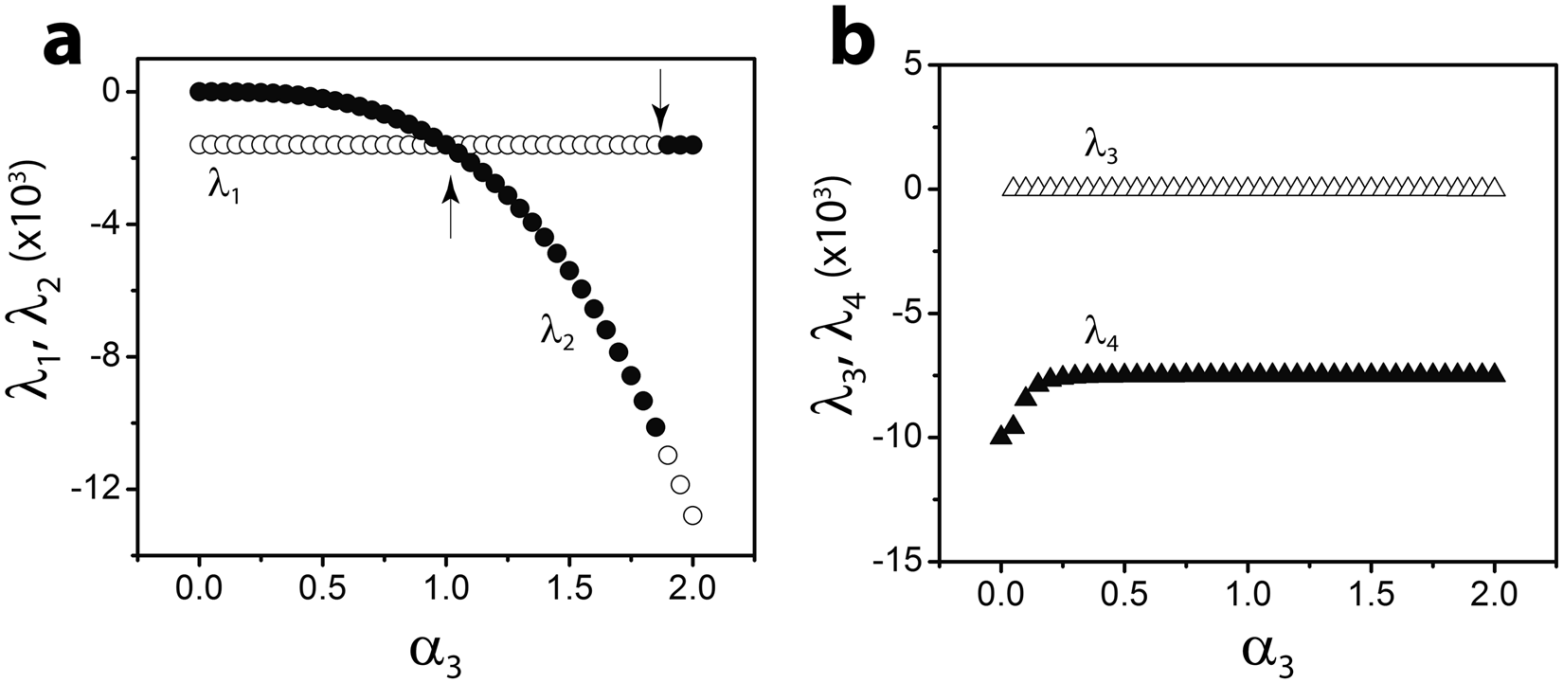
The two panels in this figure depict the eigenvalues of the ROM Jacobian matrix **B** as a function of *FA* concentration, denoted by the parameter *α*_3_. Case (**a**) shows the eigenvalues of the monomeric species *B*_1_ and ${B}_{1}^{^{\prime} }$ as a function of *α*_3_, which reveals multiple *switching* in the strength of the stability of the two species. Panel (b) shows the eigenvalues of the species *B*_*n*_ and ${B}_{n}^{^{\prime} }$. Here both the oligomers maintain their relative stability profile for all *α*_3_. Note that *B*_*i*_s and ${B}_{i}^{^{\prime} }{\rm{s}}$ refer to the normalized versions of *A*_*i*_s and *A* ^′^_*i*_s, for *i* = 1, *n*.

Based on the results of our analysis presented in the *Methods* section, Fig. 2 shows the eigenvalues *λ*_*i*_ (*i* = 1–4), of the Jacobian matrix for the mass action kinetic equations (18–21). These eigenvalues appear as a function of *α*_3_, a normalized parameter which depend on the CMC and rate constants. The eigenvalues depict the rate of decay of any perturbation to the stable, non-zero equilibria of the system of equations (18–21). It so happens that the critical parameter *α*_3_ is key to determining whether the on-pathway or off-pathway is dominant. The Fig. 2 shows some significant events in the dynamics of this system. As *α*_3_ is increased from zero, the real part of the eigenvalues *λ*_*i*_ are all negative, indicating that the equilibria are stable. When *α*_3_ < 1.0, we observe that *λ*_2_ < *λ*_1_. However, when *α*_3_ > 1.0, it follows that *λ*_2_ > *λ*_1_ indicating an exchange in the magnitude of the stability. For *α*_3_ > 1.0, the species *B*_1_ (dimensionless version of *A*_1_) dominate in magnitude and also become more stable than ${B}_{1}^{^{\prime} }$ (dimensionless version of ${A}_{1}^{^{\prime} }$), indicating a *transcritical-like bifurcation* at *α*_3_ = 1.0.

**Figure 3:**
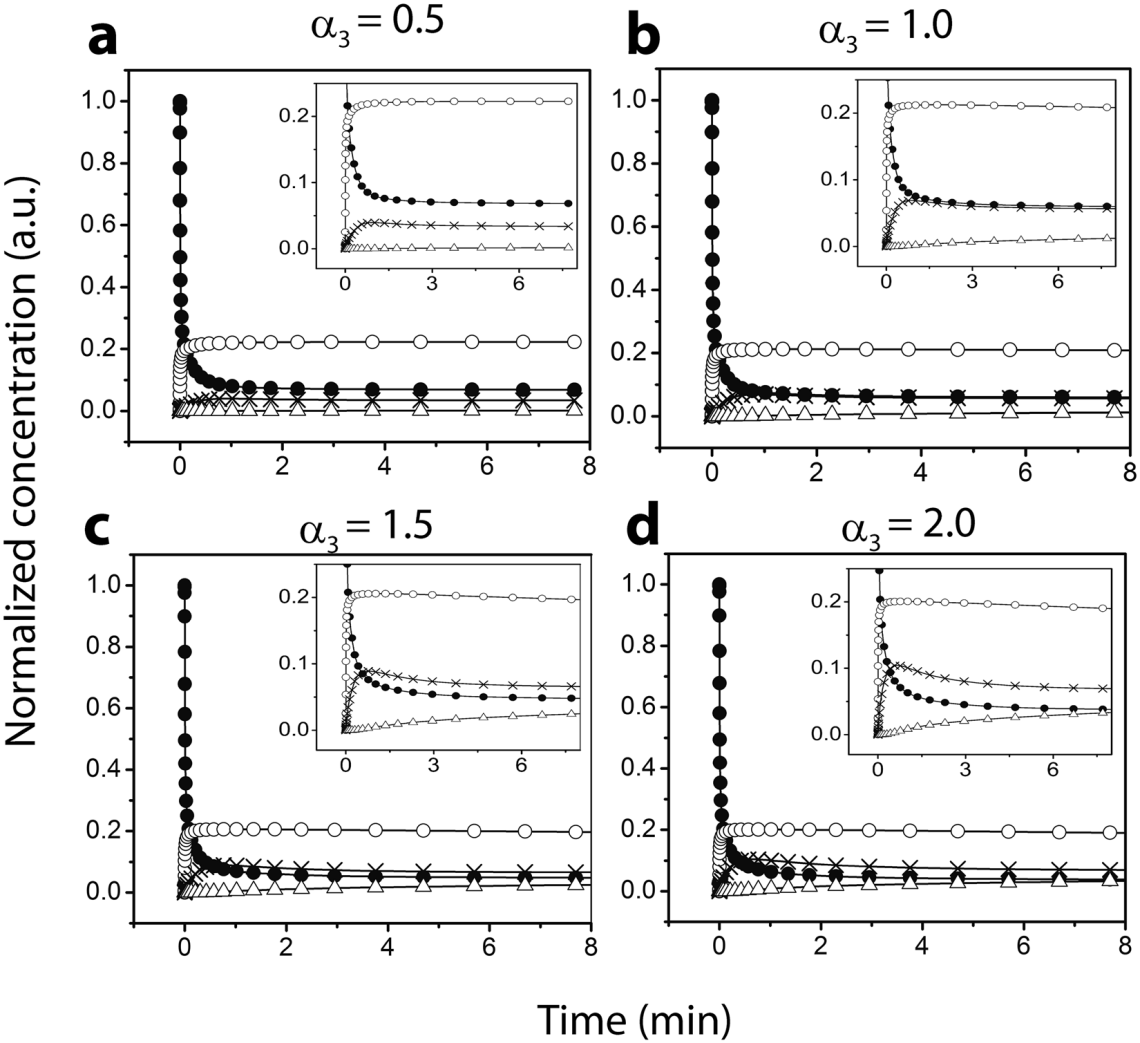
The figure depicts the population of all four species, monomers and oligomers, in the ROM as a function of time. In its initial state, only the on-pathway monomers (*B*_1_) are taken to exist which reveals itself in the declining trend of *B*_1_s and an increase in the other species over time. The evolution of the monomers and aggregates is depicted for four different values of ${\alpha }_{3}=\frac{{k}_{1}^{+}\,FA}{{k}_{1}^{-}}$ while the remaining parameters in these cases correspond to the baseline case *α*_1_ = *β*_1_ = 0.1, *α*_2_ = *β*_2_ = 100 and *n* = 4. The inset shows a zoomed in version of the same plot to clearly show the initial phase of evolution. In each of the figures, the black filled circles represent *B*_1_, the open circles represent ${B}_{1}^{^{\prime} }$, The x-shaped symbols corresponds to *B*_*n*_ and open triangles, to ${B}_{n}^{^{\prime} }$.

Note that in the dynamical systems literature, *transcritical bifurcation* refers to a stable equilibrium and an unstable equilibrium which exchange stabilities with respect to some parameter^47^. In our model system, the switching of stabilities is revealed by *α*_3_, which, by its definition, contains the ratio of the forward to backward rate constants for this reaction, as well as the fatty acid concentration which enhances the formation of the off-pathway species. Figure 3(b) shows the rate of reformation of the species *B*_*n*_ and ${B}_{n}^{^{\prime} }$, which is slower than the monomeric species.

Another interesting phenomenon that occurs in this system is a discontinuity in the eigenvalues or second switching which occurs at around *α*_3_ = 1.87 (see Fig. 3(a)). The Table 1 in the Supplementary Information captures the variety of switching behaviors and changes in behavior of the ROM system when different parameters (rate constants) are varied. At this stage, the biophysical implication of this second critical point is unclear to us although it is most certainly a symptom of the high nonlinearity of the system.

**Table 1:** Size difference between *FA*_*pm*_ and *FA*_*m*_ for different carbon chain lengths of *FA*s determined by dynamic light scattering.

Carbon chain lengths of fatty acids	diameter (nm) at above-CMC range	diameter (nm) at near-CMC range
C9	2	14–16
C10	2.7	13.5–19
C11	3.2	16–22.5
C12	3.8	19–27

We explore the effect of *n* on our system, for 1 ≤ *n* ≤ 20. Our analysis reveals significant changes to the stability of the system as *n* varies; in particular, the eigenvalues *λ*_1_ and *λ*_2_ show some dramatic changes with *n*. For 1 ≤ *n* < 12, multiple switching points (at least two) were observed with *λ*_2_ eventually being more negative than *λ*_1_, i.e. off-pathway eventually becomes more stable for sufficiently large *α*_3_. However, for 12 ≤ *n* ≤ 20, the on-pathway eventually becomes more stable. The physical significance of the multiple switching, in particular the one beyond *α*_3_ = 1 still eludes us and it remains to be verified if this an indication of some underlying bifurcation in the system or is simply an artifact of the nonlinear system or an indication of some nucleation type event observed in our previous work using similar methods^48^.

The results of our stability analysis with support from the Nash equilibrium for this system (see Methods section), allows us to draw the following conclusions: (i) The simplified, though highly nonlinear system of ROM equations with 4-species captures the essential dynamics of the A*β* system and can help explain the switching dynamics considering only the CMC; (ii) The non-dimensional parameter *α*_3_ is seen to be the key control parameter in this analysis indicating that *FA* concentration, along with the ratio of the rate constants along this “bridge” reaction pathway determine the ultimate fate of the system. (iii) The presence of a second, discontinuous switching of eigenvalues at *α*_3_ ≥ 1.87 potentially indicates a different biophysical phenomenon occurring along the off-pathway implicating the involvement of additional species that may play an important role in the *FA* dynamics. To explore the dynamics between on- and off-pathways of A*β* aggregation, we then conducted a more detailed ensemble simulation using chemical kinetics under varying concentration of *FA*s.

### Ensemble kinetic simulation (EKSs) on A*β*-*FA* dynamics

In order to further investigate the dynamics between the on- and off-pathways in the presence of *FA*s, we used the EKS approach. EKS approach involves numerical ordinary differential equation (ODE)-based simulations that encompasses ensembles of the dynamic species involved in the reaction flux. Fundamental aspects of EKSs were used in the on-pathway model that we had previously reported^10, 48^. In this report, we have expanded the EKS model to simulate various phase-transition regimes of *FA*s and A*β*, especially along off-pathway (elaborated in the Methods section). Before modeling on- and off-pathway dynamics, it is imperative to simulate the phase-transitions of *FA*s during micelle formation. We specifically chose three concentration regimes at which *FA*s show distinct phase changes: low concentrations where the *FA*s are non-micellar (*FA*_*n*_), near their CMCs where they are pseudo-micellar (*FA*_*pm*_), and high concentration where they are fully micellar (*FA*_*m*_). We conjecture the nomenclature, *pseudo-micelles* to define the dynamic state between a non-micelle and a micelle, which seemed to affect A*β* in a distinct way^22^. The hydrodynamic radii (Rh) estimates of *FA*_*pm*_ and *FA*_*m*_ indicated that the former is ~6–7 times larger than the latter, confirming the loosely-held, amorphous state of *FA*_*pm*_ (Table 1).

#### Dynamics in non-micellar phase - A*β*–*FA*_*n*_ interactions

**Figure 4:**
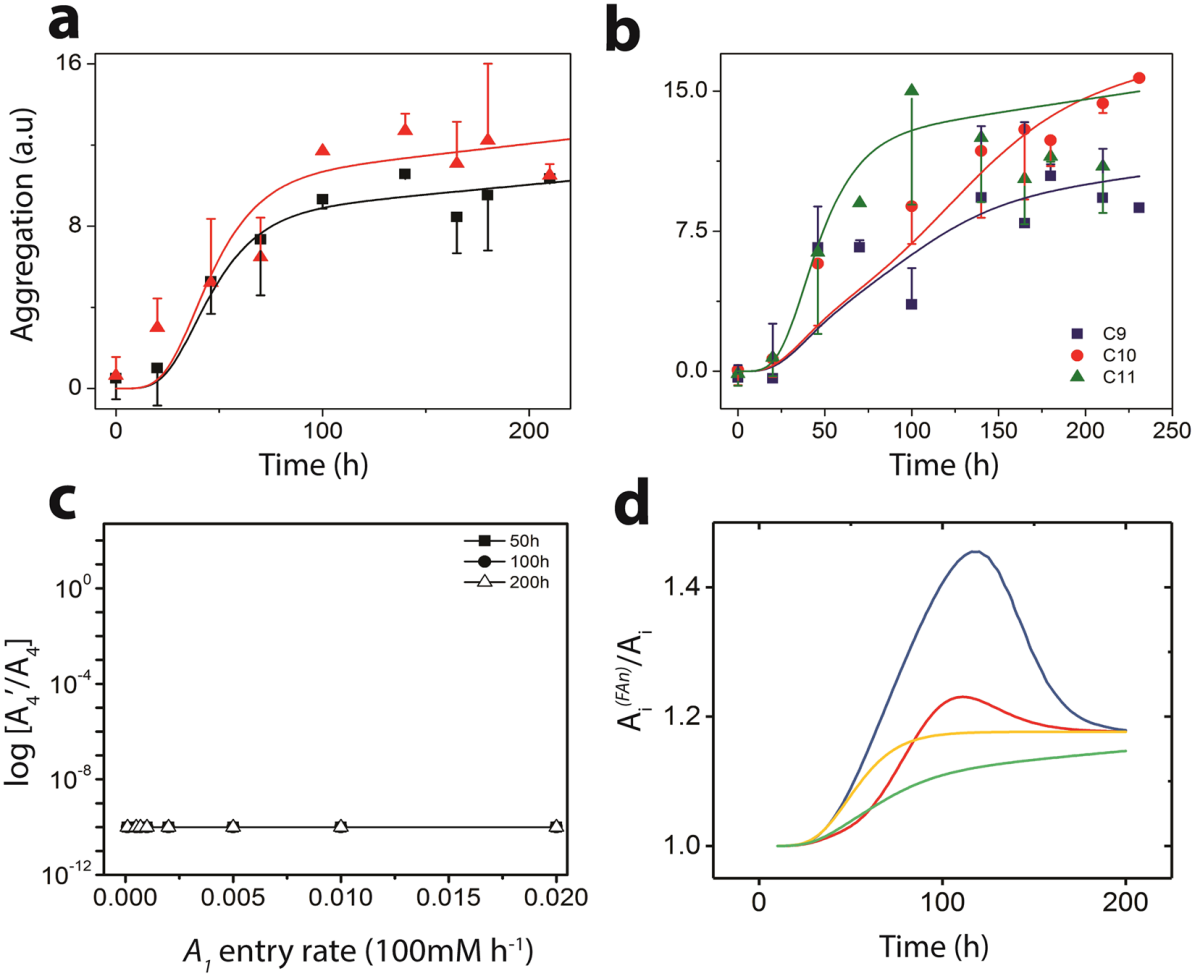
Experimental and simulated data at control and below CMC concentration. (**a**) For C12 FAs; Black squares: Experimental ThT intensity for on-pathway; Black line: simulated ThT intensity for on-pathway; Red triangles: Experimental ThT intensity for below-CMC set-up; Red line: simulated ThT intensity for below-CMC set-up; (**b**) Below-CMC experimental ThT intensity and simulated intensities for C9-C11; (**c**) Below-CMC semi-log concentration ratio for ${A}_{4}^{^{\prime} }/{A}_{4}$, i.e., the 4-mers from off-pathway and on-pathway respectively considering monomer entry rates; the oligomer concentration were recorded at 50^*th*^, 100^*th*^ and 200^*th*^ hours for C12 fatty acids; (**d**) Oligomer concentration ratios between below CMC (${A}_{i}^{F{A}_{n}}$) and on-pathway (*A*_*i*_) set-ups designated by ${A}_{i}^{F{A}_{n}}/{A}_{i}$; Red: (*A*_1_ at below-CMC)/(*A*_1_ at control); Blue: (*A*_4_ at below-CMC)/(*A*_4_ at control); Yellow: (*A*_8_ at below-CMC)/(*A*_8_ at control); Green: (*A*_12_ at below-CMC)/(*A*_12_ at control).

First, for the control experiment in the absence of *FA*s (on-pathway reactions, exclusively), we find the forward rate constants during the nucleation stage (*k*_*nuon*_) is considerably lower than the forward rate constant (*k*_*fbon*_) of elongation stage, as expected (Fig. 4(a)). The simulated data showed good correlation with the experimental data with an *R* ^2^ value of 96.2, and an equally good agreement in the lag times (Fig. 4(a)) for C12, as well as C9-C11 *FA* data (Fig. 4(b)). The estimated rate constants for control, i.e., on-pathway, are shown in Supplementary Table 2 in Appendix-B under Supplementary Information. Secondly, in the presence of *FA*_*n*_, on-pathway reactions still dominate due to the absence of micelles (or pseudo-micelles) in the solution. We hypothesize that *FA*_*n*_s interact with A*β* and catalyze aggregation by altering the rate constants along the on-pathway by a factor of *K*, resulting in a change in the final fibril concentration. The corresponding set of reactions for this model are shown in Appendix-C under Supplementary Information. We obtain the value of *K* < 1 for the best fit with the experimental data pointing to a slowdown in the elongation stage of the on-pathway (Supplementary Table 3 in Appendix-C under Supplementary Information).

The simulations of A*β* interactions with C12 *FA*_*n*_ when compared to the on-pathway fibril growth characteristics, provided two important observations. First, the overall aggregation was augmented with the introduction of *FA*_*n*_, consistent with the overall ThT intensity observed in experiments (Fig. 4(a); red). Second, the third phase of fibril growth curve (i.e., the slower growth phase towards saturation subsequent to the exponential growth phase) was delayed for a considerable amount of time with C12 *FA*_*n*_ (as opposed to the on-pathway), despite no change in the lag time (Fig. 4(a)). We hypothesize these effects are caused by the introduction of *FA*_*n*_, which modulate the on-pathway rate constants. To test this hypothesis, we systematically analyzed the effects that *FA*_*n*_ may have on the rates of on-pathway reactions at critical times of the aggregation process. First, the forward rate constants during the pre-nucleation stage was altered (increased/decreased) by a factor of *K*, which did not fit the fibril growth curve for the C12 *FA*_*n*_ case and concomitantly could not validate this behavior (data not shown). Next, upon varying the forward elongation rate constant (*k*_*fbon*_), a small decrease in the forward elongation rate constant render higher ThT intensity with delayed saturation time, and without changing the lag time. This suggests that the addition of *FA*_*n*_ molecules effectively decrease the forward elongation rate constants. According to the Lee’s and our own on-pathway models^48, 49^, the elongation stage (ie. ${A}_{12}+{A}_{1}\leftrightarrows {A}_{12}$; ${A}_{12}+{A}_{2}\leftrightarrows {A}_{12}$; … and ${A}_{12}+{A}_{11}\leftrightarrows {A}_{12}$) does not lead to the increase in ThT intensity directly as these reactions may not increase the number of ThT binding sites significantly to augment fluorescence intensities. Hence, a decrease in the elongation rate constant essentially extends the duration of the pre-nucleation stage, although without increasing the lag time. This subsequently could increase the formation of pre-nucleated on-pathway aggregates (specifically 12 mers, i.e., *A*_12_s) resulting in higher ThT intensity/fibril growth. The increased number of *A*_12_s resulting from these reactions then react with one another as well as the on-pathway oligomers during the pre-nucleation stage (monomers to 11 mers, i.e., *A*_1_–*A*_11_) to generate higher order aggregates with concomitant higher ThT intensities. The predicted *K* value for different initial *FA*_*n*_ concentrations is shown in Supplementary Table 3 in Appendix-C under Supplementary Information. Similar corresponding plots for ThT vs simulations for C9-C11 *FA*_*n*_ are shown in Fig. 4(b). The high *R* ^2^ values obtained for the fits (Supplementary Table 3) suggest that *FA*_*n*_s promote on-pathway fibrils by a slightly different mechanism as compared to the A*β* aggregation in the absence of *FA*s. Hence, as shown in Fig. 4(c), the ratio of ${A}_{4}^{^{\prime} }$/*A*_4_ is effectively zero.

We further consider two hypothetical but physically relevant scenarios in which varying rates of entry of A*β* monomers and *FA*s were considered as opposed to a fixed concentration^50^. The reaction models for this scenario were then minimally modified to consider these additional entry rates as variables to evaluate the oligomer concentration dynamics. As expected in Fig. 4(c), the interaction of *FA*_*n*_ with A*β* generated no off-pathway oligomers due to the absence of micelles or pseudo-micelles. Only on-pathway aggregates were formed exclusively, although by a slightly different mechanism. Based on these results, we determine that *FA*_*n*_s decrease the rates during the fibril elongation stage along the on-pathway, which seems to hold good for each type of *FA* used (C9-C12).

In (Fig. 4(d)), we report the oligomer ratios (of the same size) between the control and *FA*_*n*_ set-ups. We considered four oligomers for this test: *A*_1_, *A*_4_, *A*_8_, *A*_12_; in each case, these ratios rise above the value of 1, showing that the presence of more below-CMC oligomers than on-pathway oligomers in the system as time increases. Note that these were generated from two stand-alone simulations, one for the control and the other one for *FA*_*n*_ as in both cases only on-pathway oligomers are produced. The results clearly suggest that the *FA*_*n*_ scenario produces more oligomers (and possibly more fibrils) with time which supports the experimental observation of increased ThT intensity for *FA*_*n*_. Additionally, as expected the ratios are smaller for larger oligomers; this is simply because of the reaction order as fewer oligomers of larger size are formed in the system. Thus our reaction hypothesis of a slowdown of the elongation stage in the presence of *FA*_*n*_s, although counter-intuitive, accurately validates the experimental observations of increased ThT intensity.

#### Dynamics in pseudo-micellar phase - *Aβ*/*FA*_*pm*_ interactions

*FA* concentration around the CMC increases the concentration of *FA*_*pm*_ in the bulk solution, the presence of which shifts the reaction flux towards predominantly off-pathway as established experimentally^22, 51^ (Fig. 1). As the forward elongation rate constant becomes crucial only at the later phase of the on-pathway reactions, the effect of *K* was neglected here. This allowed us to assume that effectively all *FA*s convert to *FA*_*pm*_s and thus making the effects of free *FA*s during the on-pathway elongation stage negligible. The predicted rate constants from the control experimental data were used here for the on-pathway set of reactions, and the parameter space was iterated to only estimate the rate constants involving off-pathway reactions. Thus, our combined on- and off-pathway model was able to explain all the experimental observations in the presence of *FA*_*pm*_ (corresponding *R* ^2^ values are reported in Table 2 for C9-C12 *FA*s). Furthermore, previous reports identified that 12–24 mers (LFAOs) are the predominant species generated in the presence of C12 *FA*_*pm*_^22, 51^. Our simulations predict the presence of multiple conformers within the oligomer of a specific size (strains) as observed before^52^. The 12–24 mer LFAOs show conformational dynamics (represented as ${F}_{1}^{^{\prime} }$ and ${F}_{1}^{^{\prime\prime} }$ here), wherein the ${F}_{1}^{^{\prime} }$, and not ${F}_{1}^{^{\prime\prime} }$, is able to associate with itself. Note that both species (${F}_{1}^{^{\prime} }$ and ${F}_{1}^{^{\prime\prime} }$) correspond to 12–24 mer LFAOs and we considered up to four-fold lateral associations between them to form ${F}_{4}^{^{\prime} }$ which are essentially 48–96 mers as previously established^52^. Such ${F}_{4}^{^{\prime} }$ LFAOs then undergo a secondary fragmentation to produce four ${F}_{1}^{^{\prime\prime} }$ which are not allowed to laterally associate thereby making the 12–24 mers as the predominant species. This is due to subtle yet distinct structural differences between the two 12–24 mer LFAOs^53^. In our model, we identified that lateral association and fragmentation are both critically important in *Aβ*/*FA*_*pm*_ reaction dynamics, which ultimately explains the formation of stable oligomers of size 12–24 i.e., ${F}_{1}^{^{\prime\prime} }$. The predicted concentration of pseudo-micelles are shown in Table 2 while the predicted rate constants and shown in Supplementary Table 4 in Appendix-D under Supplementary Information.

**Table 2:** Relation of CMC, deviation from CMC with Experimental Fatty acid concentration, and predicted pseudo-micelle concentration; additionally reported are the *R* ^2^ fit between simulation and experimental ThT intensity.

Fatty acid	CMC (*mM*)	Experimental concentration in (*mM*) from ref. 22	*R* ^2^	Predicted pseudo-micelle concentration (*μM*)
C12	7	5	95.5	11
C11	22	20	92.5	16
C10	50	50	88.8	45
C9	85	100	91.1	8

**Figure 5:**
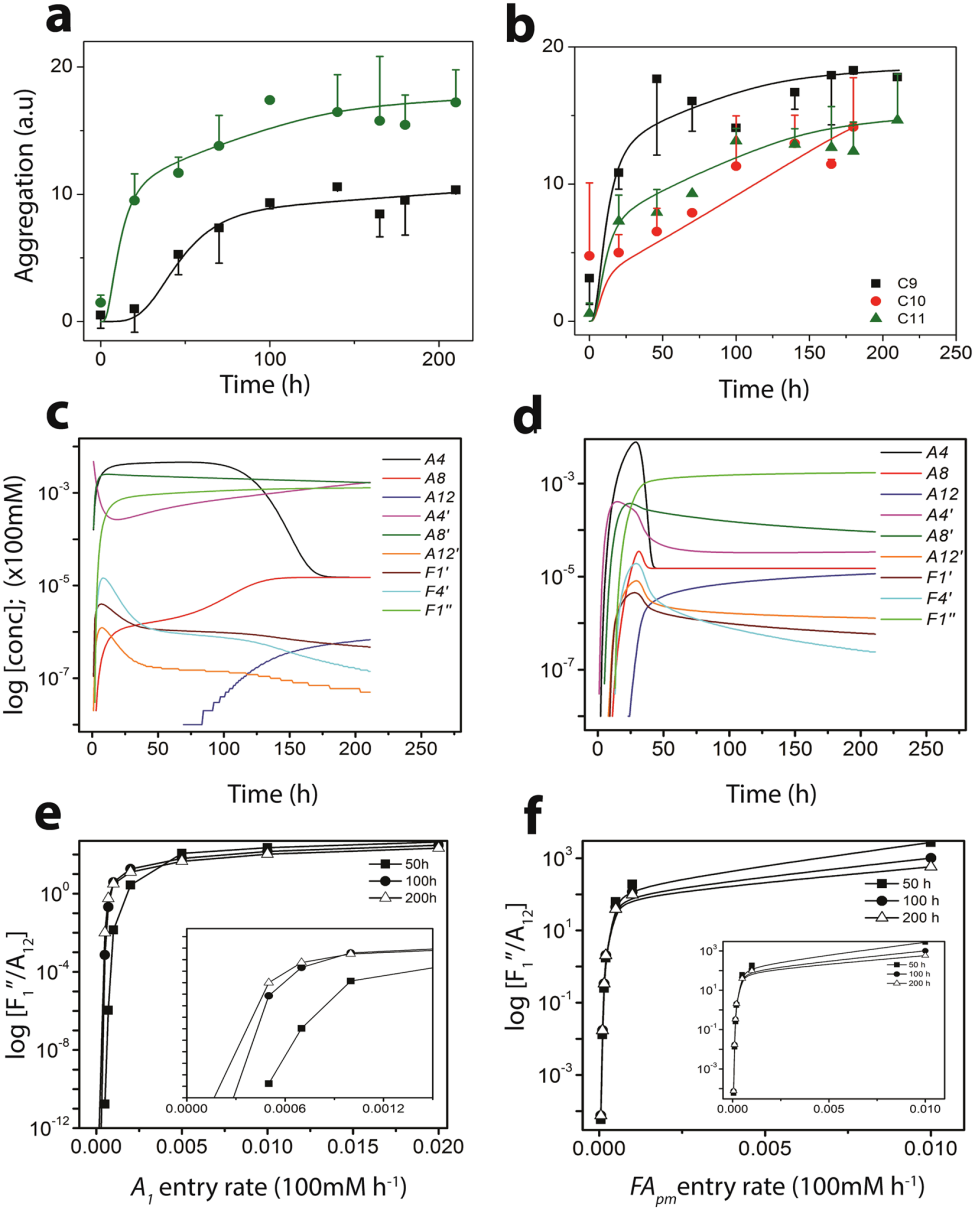
Experimental and simulated data at near CMC concentration: (**a**) and (**b**). Comparisons of the concentration of important oligomers between on- and off-pathways considering monomer, pseudo-micelle and micelle entry rates into the system: (**c**–**f**); the oligomer concentration were recorded at 50^*th*^, 100^*th*^ and 200^*th*^ hours for C12 fatty acids. (**a**) Green circles: Experimental ThT intensity for near-CMC set-up; Green line: simulated ThT intensity for near-CMC set-up; Black squares: Experimental ThT intensity for on-pathway; Black line: simulated ThT intensity for on-pathway; (**b**) Near-CMC experimental ThT intensity and simulated intensities for C9-C11; (**c**) Oligomer concentration plot for C12 fatty acids at the near-CMC range with 25 *μ*M monomers (i.e., *A*_1_); (**d**) Oligomer concentration for C12 fatty acids at the near-CMC range considering 0.1 *μ*M/h rate of entry of *A*_1_ into the system; (**e**) Near-CMC semi-log concentration ratio for ${F}_{1}^{^{\prime\prime} }/{A}_{12}$, i.e., the structurally distinct 12-mers from off-pathway undergoing secondary fragmentation and the 12-mers from on-pathway respectively considering monomer entry rates; (**f**) Near-CMC semi-log concentration ratio for ${F}_{1}^{^{\prime\prime} }/{A}_{12}$, considering pseudo-micelle (denoted by *FA*_*pm*_) entry rates.

**Figure 6:**
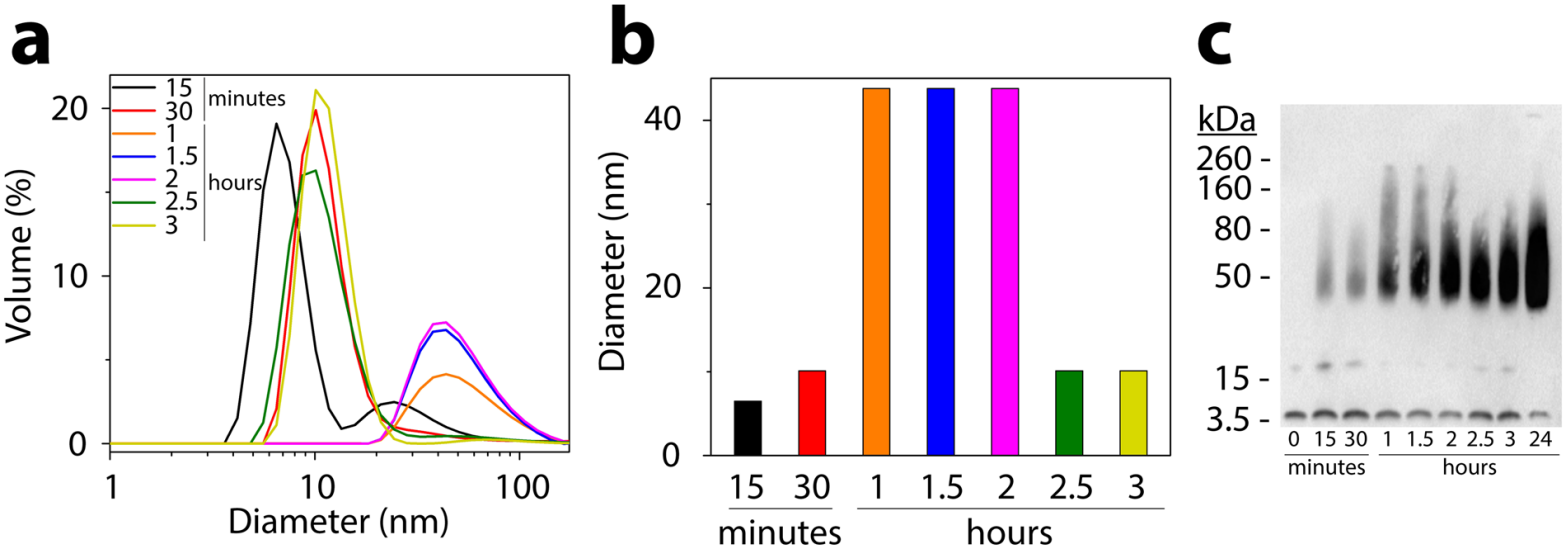
Dynamics of A*β* interaction with C12 FA. (**a**) Size of the aggregates estimated by DLS after 15 (black) and 30 (red) minutes of incubation at 37 °C, along with data after 1 (orange), 1.5 (blue), 2 (pink), 2.5 (green) and 3 (yellow) hours of incubation, respectively. (**b**) Variations in diameter (nm) for each time point, calculated from data in panel (a). (**c**) SDS-PAGE with immunoblotting of LFAO formation at 37 °C from 0 to 24 hours, respectively. The amount loaded into each lane was kept constant at 1.1 *μ*g.

Overall simulation of the ThT intensity data for *FA*_*pm*_ regime yielded good correspondence with the experimental results for C12 (Fig. 5(a); green). We obtained a high *R* ^2^ value and nearly similar correspondence with the experimental lag time and saturation time for C12 as well for C9-C11 *FA*s (Fig. 5(b)). The comparative concentration dynamics of off-pathway and on-pathway aggregates in the presence of *FA*_*pm*_ are shown in Fig. 5(c–f) for C12 fatty acid. Figure 5(e) is the data for constant *FA*_*pm*_ and varying A*β* monomer entry rates while Fig. 5(f) is the data obtained for constant monomers and varying *FA*_*pm*_ entry rates. In our model, for the sake of simplicity and comparison, we considered aggregates of similar sizes from both on- and off-pathway. We observe that the on-pathway oligomers *A*_4_, *A*_7_ and *A*_12_ show delayed emergence (Fig. 5(c)). It is noteworthy that *A*_4_ emerges rapidly, and saturates before decreasing in concentration. This behavior is due to the elongation of oligomers by *A*_12_s that occurs along the on-pathway, which form larger fibrils at the cost of *A*_4_ consumption. Along the off-pathway, aggregates ${A}_{4}^{^{\prime} }$, ${A}_{8}^{^{\prime} }$ and ${A}_{12}^{^{\prime} }$ show expected characteristics of being formed rapidly initially before saturating (Fig. 5(d)). It is important to note that the elongation in the off-pathway leads to ${F}_{1}^{^{\prime} }$ consuming ${A}_{4}^{^{\prime} }$ and ${A}_{12}^{^{\prime} }$ oligomers to form the 12–24 mers as discussed previously. Such ${F}_{1}^{^{\prime} }{\rm{s}}$ then associate to form ${F}_{4}^{^{\prime} }$ which are seen in greater quantities towards the beginning with their concentration decreasing with time, which is likely due to fragmentation of high molecular weight aggregates. Finally, most of the *FA*_*pm*_ dynamics leads to the formation of the ${F}_{1}^{^{\prime\prime} }$ with time, which becomes the predominant ThT positive species in the system. This dynamics is further corroborated by incorporation of physiologically relevant monomer entry rates into the system (Fig. 5(d)). Here, the dynamics show more conclusively the dominance of the ${F}_{1}^{^{\prime\prime} }$ oligomer in the system with increasing time followed by ${A}_{8}^{^{\prime} }$ and ${A}_{4}^{^{\prime} }$. In order to verify the potential fragmentation identified by our simulation, we investigated this possibility experimentally by incubating A*β* and C12 *FA* near its CMC (Fig. 6). The results indicate that incubation rapidly results in the formation of aggregates corresponding to 50–60 nm diameter within one hour of incubation (Fig. 6(a) and (b)). However, after two hours of incubation, the size of the aggregates decreased to 10 nm diameter. No more change in the aggregate size was observed even after 48 h (data not shown). This phenomenon was also observed in the aggregate size monitored by immunoblotting (Fig. 6(c)), and supports the possibility of fragmentation of aggregates as predicted by the EKS model predictions.

Figure 5(e) and (f) show the ratio of the oligomer distributions for C12 fatty acids considering constant entry rates of A*β* monomers with fixed initial *FA*_*pm*_ concentration (Fig. 5(e)) and *FA*_*pm*_ into the system for fixed initial monomer concentration (Fig. 5(f)), respectively, as described before for *FA*_*n*_. These plots illustrate the switching behavior between the on- and off-pathways as predicted by the ROM stability analysis (Figs 2 and 3). For example, we can observe that for negligible monomer or *FA*_*pm*_ entry rates, the on-pathway is active, but the switching occurs quickly to off-pathway when the monomer entry rate is about 0.001 × 100 *μ*M *h* ^−1^ (Fig. 5(e)). This switching effect is however faster when the *FA*_*pm*_ entry rate is considered as shown in Fig. 5(f) (the amplitude of the ${F}_{1}^{^{\prime\prime} }/{A}_{12}$ ratio is higher in the latter case). Also, the ratios are higher after 50 h of incubation as compared to after 100 or 200 h (Fig. 5(e) and (f)). This suggests that either the ${F}_{1}^{^{\prime\prime} }$ concentration decreases with time or the *A*_12_ increases with time or both cases happen simultaneously. As shown in Fig. 5(d), the increase in *A*_12_ is more pronounced that that in ${F}_{1}^{^{\prime\prime} }$ concentration with time (note that both concentration increase with time) resulting in a decrease in the recorded ratio with time. Nevertheless, we can conclude that generally the switch to off-pathway is extremely fast with higher *FA*_*pm*_ entry rates being a more crucial factor than an increase in the monomer entry rates for the observed effect.

Comparing the *FA*_*pm*_ ThT intensity plots (Fig. 5(b)), we additionally observe that the concentration of *FA*_*pm*_ significantly affects the behavior of aggregation. For example, higher the *FA*_*pm*_ concentration present in the system (correlates with smaller fatty acid chain length), more the saturation is delayed for the ThT growth curve. As expected, our predicted *FA*_*pm*_ concentration is highest near the CMC, and the concentration starts to decrease when total *FA* concentration is either increased or decreased. The predicted *FA*_*pm*_ concentration for each fatty acid chain length at the near CMC zone experiments is shown in Table 2. Note that the prediction for the C9 case shows a small deviation primarily because the experiment was done at a 100 mM concentration while the actual CMC was 85 mM, i.e., a deviation of 17.6%.

#### Dynamics in micellar phase - *Aβ* − *FA*_*m*_ interactions

**Figure 7:**
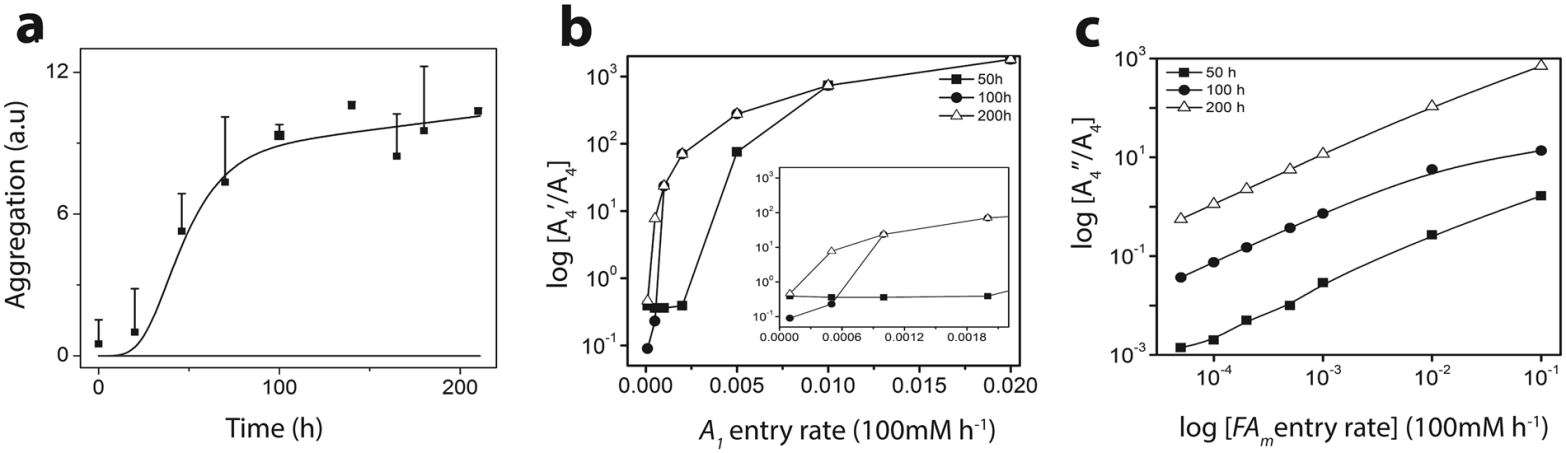
Experimental (points) and simulated (lines) ThT intensities at (**a**) Above-CMC concentration; Black: on-pathway; Blue line: simulated ThT intensity at above-CMC set-up. (**b**) Above-CMC semi-log concentration ratio for ${A}_{4}^{^{\prime} }/{A}_{4}$, i.e., the 4-mers from off-pathway and on-pathway respectively considering monomer entry rates; (**c**) Above-CMC semi-log concentration ratio for ${A}_{4}^{^{\prime} }/{A}_{4}$, considering micelle (denoted by *FA*_*m*_) entry rates.

The above-CMC regime (*FA*_*m*_) only produces ${A}_{4}^{^{\prime} }$ along the off-pathway that do not seem to aggregate any further due to the stabilization by the micelles^22^ (Fig. 7(a)). Therefore, the corresponding reaction model only considers the pre-nucleation stage in the off-pathway. Furthermore, the ${A}_{4}^{^{\prime} }$ oligomers do not bind to ThT and hence, are invisible to this assay. This complicates the precise predictions of ${A}_{4}^{^{\prime} }$ concentrations and direct correlation with the experimental data. Therefore, we could not estimate the accurate rate constants for *FA*_*m*_. It is noteworthy that we assumed the off-pathway reactions in the presence of *FA*_*pm*_ also undergoes similar pre-nucleation stage reactions besides others. The rate constants of the pre-nucleation stage from *FA*_*pm*_ data was used for this simulation to determine the dynamics of ${A}_{4}^{^{\prime} }$ production. As shown in Fig. 7(b) and (c), one could observe that ${A}_{4}^{^{\prime} }$ is generated rapidly accompanying a switching to the off-pathway, even with negligible monomer or micelle entry rates. As the ${A}_{4}^{^{\prime} }$ formation presumably involves a concerted single step reaction, the concentration profile is different from its on-pathway counterpart, *A*_4_. Specifically, as micelle entry rate increases, number of micelles in the system also increases such that the ${A}_{4}^{^{\prime} }$ concentration increases much faster than that of the *A*_4_ concentration as shown in Fig. 7(c). However, Fig. 7(c) suggests that for a fixed initial micelle concentration, and as the A*β* monomer entry increases, the ratio of ${A}_{4}^{^{\prime} }/{A}_{4}$ goes towards saturation. This is potentially due to reduction in the number of micelles available for accommodating A*β* on the micellar surface.

## Discussion

The results reported here by two independent methods (ROM and EKS) reaffirm the experimental observations that *FA*s modulate A*β* aggregation. Specifically, the following significant conclusions can be drawn from this work: (a) interactions of A*β* with non-micellar *FA*s (*FA*_*n*_) generate predominantly high molecular weight, on-pathway aggregates. (b) Interactions of A*β* with *FA*s near the latter’s CMC (*FA*_*pm*_) generate predominantly low molecular weight oligomers (12–24 mers) along off-pathway along with some minor quantities of on-pathway fibrils. (c) Interactions of A*β* with high concentrations of *FA*s (*FA*_*m*_; above their CMCs) exclusively generate off-pathway 4–5 mer oligomers without any fibrils suggesting that the on-pathway was completely turned off.

Perhaps most significantly, simulations from both ROM and EKS, as well as the previously demonstrated experimental evidence have identified that both concentration and phase-transitions of *FA*s dictate switching of A*β* aggregation pathways. Furthermore, the report demonstrates that under three distinct phases, *FA*_*n*_, *FA*_*pm*_, and *FA*_*m*_, the *FA*s not only modulate A*β* aggregation pathways but also promote low molecular weight oligomers, which are increasingly known to be the main pathogenic species in AD and other related neurodegenerative disorders. Specifically under pseudo-micellar conditions, where *FA*_*pm*_ undergoes a distinct phase transition, they preferentially promote and stabilize low-molecular weight oligomers. The ability of certain phases of *FA*s, or in general any biological surfactants, to promote the formation of low molecular weight, off-pathway presents high significance in AD pathology. This is because oligomers generated in this manner could lead to the formation of various toxic ‘conformeric strains’ of A*β* aggregates, which could manifest in distinct phenotypic manifestations observed in AD. Indeed, Dean *et al*., have shown that LFAOs, which are generated in the presence of C12 *FA*s, are able to propagate morphologically distinct fibrils and caused excessive cerebral amyloid angiopathy (CAA) in transgenic mice brains, which was absent in similar reactions seeded with on-pathway fibrils^52^.

Mechanistically, simulations reported here have revealed a deeper understanding of A*β*-*FA* interactions. Some of the salient dynamics involved in the modulation of A*β* aggregation by *FA*s in distinct phases include: (i) in the presence of *FA*_*n*_, the free *FA*s seem to slow the kinetics of elongation (or post-nucleation) stage along the on-pathway, (ii) in the presence of *FA*_*m*_, aggregation does not proceed beyond ${A}_{4}^{^{\prime} }$ (or ${A}_{5}^{^{\prime} }$) likely due to the stabilization of A*β* by *FA*_*m*_ as A*β*-*FA*_*mm*_ complex. Further investigation of this complex by molecular dynamics based studies to infer why such complexes cannot aggregate is on-going and will be reported at a later date, and (iii) in the presence of *FA*_*pm*_, A*β* forms *A*_4_ during the pre-nucleation phase followed by a slower progression to ${A}_{12}^{^{\prime} }$, and elongation to form 12–24 mers denoted by ${F}_{1}^{^{\prime} }$. Association of ${F}_{1}^{^{\prime} }$ to form ${F}_{4}^{^{\prime} }$, which is thermodynamically unstable, leads to a fragmentation to form ${F}_{1}^{^{\prime\prime} }$ as supported by experimental evidence (Fig. 6). The most likely possibility is that the ${F}_{1}^{^{\prime\prime} }$ are structurally distinct and different from ${F}_{1}^{^{\prime} }$ that are trapped in some kinetic minimum along the off-pathway as thought to be^22, 51^, which renders them incapable of aggregating further.

**Figure 8:**
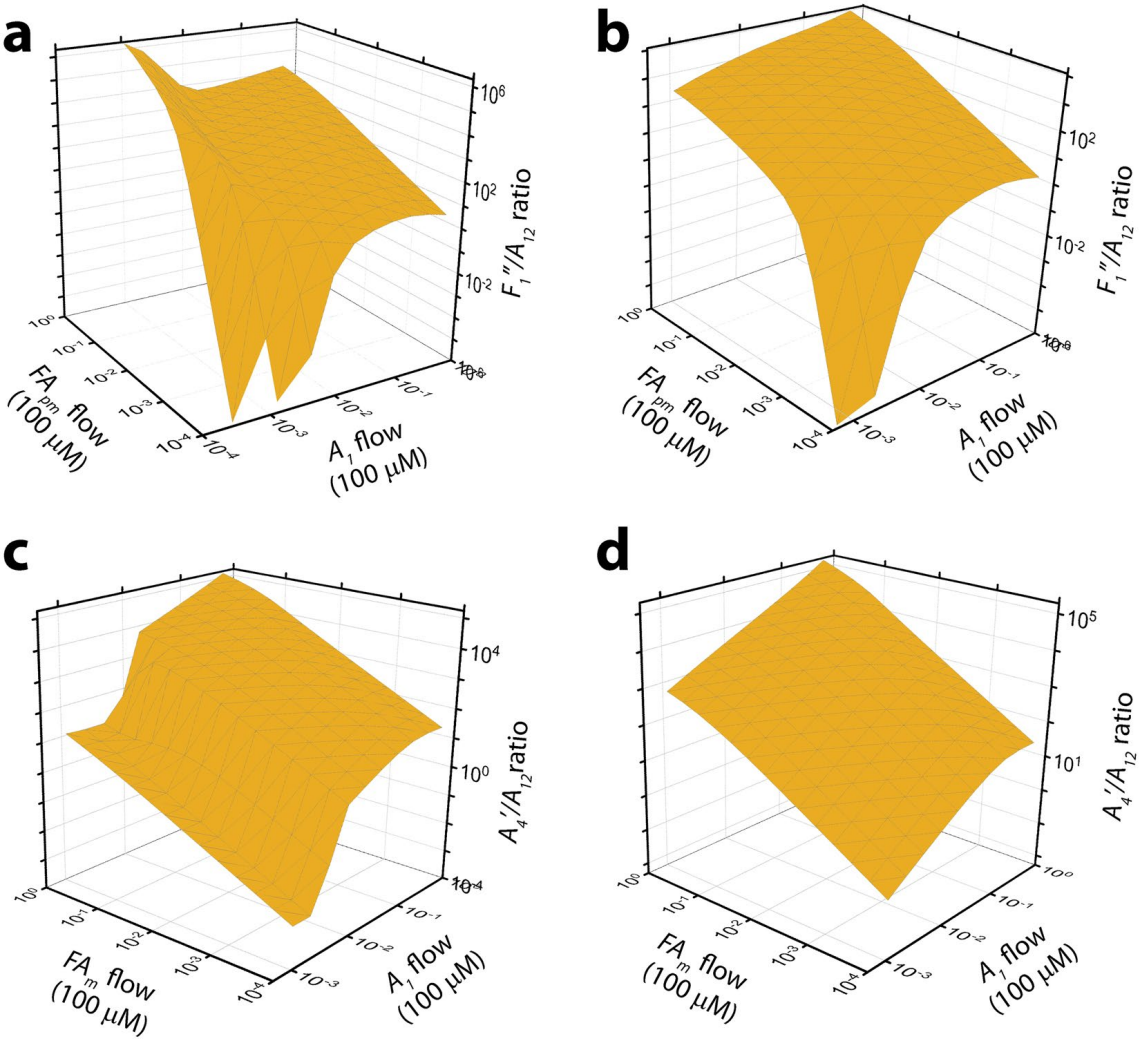
On- and Off-pathway switching dynamics at near-CMC and above-CMC zones considering *Aβ* and pseudo-micelle entry rates for C12 fatty acid. (**a**) switching dynamics at near-CMC zone at the 50^*th*^ hour; (**b**) switching dynamics at near-CMC zone at the 200^*th*^ hour; (**c**) switching dynamics at above-CMC zone at the 50^*th*^ hour; (**d**) switching dynamics at above-CMC zone at the 200^*th*^ hour.

In addition to these insights, EKS model also considered a physiological scenario in which instead of constant amount or A*β* or FAs, a constant influx of A*β* monomers as well as pseudo-micellar and micellar FAs were considered. Incorporating specific entry rates for A*β* and FAs, we observe that the presence of pseudo-micellar phase influences the switching of pathways more than that induced by the monomers. However, we observed that even a slight increase in the entry rates of either FA or A*β* is enough to switch the dynamics preferentially towards the off-pathway. This is evident in Fig. 8, which shows a surface plot which considers the monomer and *FA*_*pm*_/*FA*_*m*_ rates. The corresponding oligomer concentration ratios along off- and on-pathway show that the on-pathway is active only when *FA*_*pm*_ entry rates are very low (Fig. 8(a) and (b)). After 50 h of incubation, off-pathway is predominantly activated for the highest *FA*_*pm*_ entry rate and low monomer entry rates. In other words, all monomers entering the system are consumed by *FA*_*pm*_ leaving little scope for on-pathway reaction to occur. However, after 200 h, off-pathway is most active when both *FA*_*pm*_ and monomer entry rates are high resulting in most monomers being consumed by the *FA*_*pm*_. The scenario is bit different for *FA*_*m*_, where the off-pathway is most active when both monomer and *FA*_*m*_ entry rates are high both after 50 and 200 h (Fig. 8(c) and (d)). The dynamics seems to be more stable after 200 h than after 50 h, as oligomer concentrations saturate by then. However after 50 h, a switch to off-pathway happens only with sufficiently high A*β* monomer and *FA*_*m*_ entry rates, and there may be a critical point in this entry rate for both that would dictate whether a switch to the off-pathway occurs or not. In later times though (200 h, for example), there is a higher propensity of the system to switch to the off-pathway even with lower monomer or micelle entry rates.

Overall, this report has brought forth several interesting observations on the dynamics of A*β*-FA interactions, which seems to depend critically on the concentration and phase of FAs. Furthermore, the switching dynamics in conjunction with the constant infusion of A*β* monomers and FAs in their distinct phases illustrate the potential of biological interfaces to influence A*β* into forming several different conformationally distinct aggregates. Such conformeric strains could then influence phenotypic outcomes in AD pathology.

## Methods

### Experimental

#### Preparation of large fatty acid-derived oligomers (LFAOs)

C12:0 fatty acid (5 mM) with 50 mM NaCl was added to freshly purified A*β* monomer (50 *μ*M) buffered in 20 mM Tris, pH 8.0, and kept at 37°C in quiescent conditions. Separate reactions were started in a staggered fashion such that at the time of data collection, the sample would reach the desired time point.

#### Dynamic light scattering (DLS)

DLS data was collected using a Zetasizer Nano S instrument (Malvern, Inc., Worcestershire, UK). At each time point, DLS was collected by averaging 24 runs of 5 s each with a pre equilibration time of 30 s. This average was used to determine the diameter (nm) using the volume (%) function. For determining micelle size without A*β*, stock concentrations of C9:0, C10:0, C11:0, and C12:0 were diluted to the appropriate concentration in 50 mM NaCl buffered in 20 mM Tris, pH 8.0.

#### SDS-PAGE with immunoblotting

LFAO preparations were staggered in start such that at the time of initiating SDS-PAGE experiments, each sample was at the desired time point. Samples were diluted into 1X Laemmli loading buffer with 1% SDS, and loaded onto 4–20% BioRad TGX gels without boiling. Pre-stained MW markers (Novex Sharp Protein Standard, Life Technologies) were run in parallel for MW determination. Proteins were transferred to 0.2 *μ*m nitrocellulose membrane (BioRad) and boiled for 1 min in a microwave oven in 1X PBS, followed by blocking for 1.5 h in 1X PBS containing 5% nonfat dry milk with 1% tween 20. Blots were then probed overnight at 4 °C with a 1:6000 dilution of Ab5 monoclonal antibody, which detects amino acids 1–16 of A*β*. Blots were then incubated with a 1:6000 dilution of anti-mouse, horseradish peroxidase conjugated secondary antibody and developed with ECL reagent (Thermo Scientific).

### Reduced Order model (ROM)

The system of differential equations corresponding to Eqns 1 determines the evolution of the 4 species (${A}_{1},{A}_{1}^{^{\prime} },{A}_{n}^{^{\prime} },{A}_{n}^{^{\prime} }$) and are given by

4 $$\frac{d{A}_{1}}{dt}=n{k}_{2}^{-}{A}_{n}-n{k}_{2}^{+}{A}_{1}^{n}+{k}_{1}^{-}{A}_{1}^{^{\prime} }-{k}_{1}^{+}{A}_{1}FA$$

5 $$\frac{d{A}_{1}^{^{\prime} }}{dt}=n{k}_{3}^{-}{A}_{n}^{^{\prime} }-n{k}_{3}^{+}{A}_{1^{\prime} }^{n}-{k}_{1}^{-}{A}_{1^{\prime} }+{k}_{1}^{+}{A}_{1}FA$$

6 $$\frac{d{A}_{n}}{dt}=-{k}_{2}^{-}{A}_{n}+{k}_{2}^{+}{A}_{1}^{n}$$

7 $$\frac{d{A}_{n}^{^{\prime} }}{dt}=-{k}_{3}^{-}{A}_{n}^{^{\prime} }+{k}_{3}^{+}{A}_{1}^{^{\prime} n}$$

The above equations are better suited for analysis in non-dimensional form. If we chose the characteristic concentration and time as *A*_0_ (the equilibrium concentration of monomers) and $\frac{1}{{k}_{1}^{-}}$, respectively, then the dimensionless variables can be defined as follows:

8 $$\begin{array}{rcl}{B}_{1} & = & \frac{{A}_{1}}{{A}_{0}};{B}_{1}^{^{\prime} }=\frac{{A}_{1}^{^{\prime} }}{{A}_{0}};{B}_{n}=\frac{{A}_{n}}{{A}_{0}};{B}_{n}^{^{\prime} }=\frac{{A}_{n}^{^{\prime} }}{{A}_{0}}\\ {\alpha }_{1} & = & \frac{{k}_{2}^{-}}{{k}_{1}^{-}};{\alpha }_{2}=\frac{{k}_{2}^{+}{A}_{0}^{n-1}}{{k}_{1}^{-}};{\alpha }_{3}=\frac{{k}_{1}^{+}FA}{{k}_{1}^{-}};{\beta }_{1}=\frac{{k}_{3}^{-}}{{k}_{1}^{-}};{\beta }_{2}=\frac{{k}_{3}^{+}{A}_{0}^{n-1}}{{k}_{1}^{-}};\end{array}$$

The corresponding dimensionless differential equations, in terms of dimensionless time ‘*s*’, now can be written as

9 $$\frac{d{B}_{1}}{ds}=n{\alpha }_{1}{B}_{n}-n{\alpha }_{2}{B}_{1}^{n}+{B}_{1}^{{}^{^{\prime} }}-{\alpha }_{3}{B}_{1}$$

10 $$\frac{d{B}_{1}^{{}^{^{\prime} }}}{ds}=n{\beta }_{1}{B}_{n}^{{}^{^{\prime} }}-n{\beta }_{2}{B}_{1}^{{}^{^{\prime} }n}+{\alpha }_{3}{B}_{1}-{B}_{1}^{{}^{^{\prime} }}$$

11 $$\frac{d{B}_{n}}{ds}={\alpha }_{2}{B}_{1}^{n}-{\alpha }_{1}{B}_{n}$$

12 $$\frac{d{B}_{n}^{{}^{^{\prime} }}}{ds}={\beta }_{2}{B}_{1}^{{}^{^{\prime} }n}-{\beta }_{1}{B}_{n}^{{}^{^{\prime} }}$$

#### Equilibrium points

In the equations (4–7), if we consider the limit $({k}_{1}^{+},{k}_{1}^{-},FA)\to (0,0,0)$, then the off-pathway dynamics is turned off, leaving only the reaction

$$n\cdot {A}_{1}\underset{{k}_{2}^{-}}{\overset{{k}_{2}^{+}}{\rightleftharpoons }}{A}_{n}.$$

However, the on-pathway cannot be switched off and always persists. In this case the equilibrium solution is given by the pair $({A}_{0},{(\frac{{k}_{2}^{+}}{{k}_{2}^{-}}{A}_{0})}^{\mathrm{1/}n})$.

Turning our attention now to the more general case of the non-dimensional equations (9–12), the equilibrium points, ${{\bf{B}}}_{e}=({B}_{\mathrm{1,}e},{B}_{\mathrm{1,}e}^{^{\prime} },{B}_{n,e},{B}_{n,e}^{^{\prime} })$ can be obtained by the vanishing of the equations (9–12). Solving for these four simultaneous equations, we obtain the following relations, in terms of the equilibrium concentration *B*_1,*e*_

13 $${B}_{n,e}=\frac{{\alpha }_{2}}{{\alpha }_{1}}{B}_{\mathrm{1,}e}^{n};{B}_{\mathrm{1,}e}^{^{\prime} }={\alpha }_{3}{B}_{\mathrm{1,}e};{B}_{n,e}^{^{\prime} }=\frac{{\beta }_{2}{\alpha }_{3}^{n}}{{\beta }_{1}}{B}_{\mathrm{1,}e}^{n}\mathrm{.}$$

The subscript *e* indicates that these species are in equilibrium.

It should also be noted that if one treats the on- and off-pathway dynamics as a “competition” in accordance with the term used in game-theory^54, 55^, then the equilibrium ${{\bf{B}}}_{e}{|}_{{\alpha }_{3}=1}={{\bf{B}}}_{Nash,e}$ is nothing but the Nash equilibrium of the system given by

$${{\bf{B}}}_{Nash,e}:=({B}_{\mathrm{1,}e},{B}_{\mathrm{1,}e},\frac{{\alpha }_{2}}{{\alpha }_{1}}{B}_{\mathrm{1,}e}^{n},\frac{{\beta }_{2}}{{\beta }_{1}}{B}_{\mathrm{1,}e}^{n})$$

(see Fig. 3 for an example of such an equilibrium). This equilibrium exists only at *α*_3_ = 1 which also will be seen to have special significance in terms of the stability of the system. It follows that when *α*_3_ > 1, then the off-pathway species dominate creating more equilibrium concentrations of ${B}_{\mathrm{1,}e}^{^{\prime} }$ and for *α*_3_ < 1, on-pathway dominates resulting in greater production of ${B}_{\mathrm{1,}e}$.

We define ${k}^{+}={k}_{1}^{+}FA$ and ${k}^{-}={k}_{1}^{-}$ so that the former leads to off-pathway and the latter to on-pathway. Table 3 below points to a sample case of the various strategies in the competition between the two pathways with the pay-off for each “strategy” given by the equilibrium concentrations.

**Table 3:**
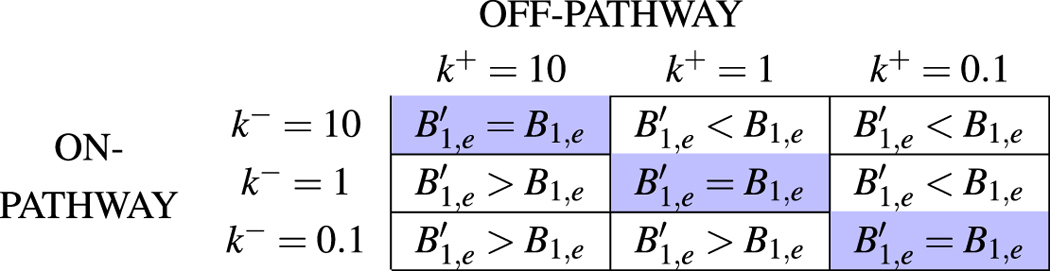
A sample game-theoretical payoff matrix to depict the on- and off-pathway competition. The cells highlighted in blue represent the Nash equilibrium.

#### Time evolution of the different species

The system of equations (4–7) were solved using the Matlab ode45 function. A sample solution is shown in Fig. 2 corresponding to initial conditions: ${B}_{1}(0)=2$, ${B}_{1}^{^{\prime} }(0)={B}_{n}(0)={B}_{n}^{^{\prime} }(0)=0$, ${\alpha }_{1}={\beta }_{1}=1$, ${\alpha }_{2}={\beta }_{2}=1$ and *n* = 4. Also, here ${\alpha }_{3}=1$ which results in ${B}_{1,e}={B}_{1,e}^{^{\prime} }$.

#### Linear stability analysis

To study the stability of the four species, we linearly perturb the system which is mathematically represented by

14 $${B}_{1}={B}_{1,e}+\varepsilon {X}_{1}$$

15 $${B}_{1}^{^{\prime} }={B}_{1,e}^{^{\prime} }+\varepsilon {Y}_{1}$$

16 $${B}_{n}={B}_{n,e}+\varepsilon {X}_{n}$$

17 $${B}_{n}^{^{\prime} }={B}_{n,e}^{^{\prime} }+\varepsilon {Y}_{n}$$

then the linearized set of differential equations for the perturbations, at *o*(*ε*) becomes

18 $$\frac{d{X}_{1}}{ds}=n{\alpha }_{1}{X}_{n}-{n}^{2}{\alpha }_{2}{B}_{1,e}^{n-1}{X}_{1}+{Y}_{1}-{\alpha }_{3}{X}_{1}$$

19 $$\frac{d{Y}_{1}}{ds}=n{\beta }_{1}{Y}_{n}-{n}^{2}{\beta }_{2}{\alpha }_{3}^{n-1}{B}_{1,e}^{n-1}{Y}_{1}-{Y}_{1}+{\alpha }_{3}{X}_{1}$$

20 $$\frac{d{X}_{n}}{ds}=n{\alpha }_{2}{B}_{1,e}^{n-1}{X}_{1}-{\alpha }_{1}{X}_{n}$$

21 $$\frac{d{Y}_{n}}{ds}={\beta }_{2}{\alpha }_{3}^{n-1}{B}_{1,e}^{n-1}{Y}_{1}-{\beta }_{1}{Y}_{n}$$

which can be expressed in operator form with the perturbation matrix, **B** given by

$${\bf{B}}=(\begin{array}{cccc}-{n}^{2}{\alpha }_{2}{B}_{\mathrm{1,}e}^{n-1}-{\alpha }_{3} & 1 & n{\alpha }_{1} & 0\\ {\alpha }_{3} & -{n}^{2}{\beta }_{2}{\alpha }_{3}^{n-1}{B}_{\mathrm{1,}e}^{n-1}-1 & 0 & n{\beta }_{1}\\ n{\alpha }_{2}{B}_{\mathrm{1,}e}^{n-1} & 0 & -{\alpha }_{1} & 0\\ 0 & {\beta }_{2}{\alpha }_{3}^{n-1}{B}_{\mathrm{1,}e}^{n-1} & 0 & -{\beta }_{1}\end{array})$$

The stability of the equilibrium is found from computing the eigenvalues, *λ*_*i*_ (*i* = 1, 2, 3, 4) of the matrix *B*. This is best done numerically. In the figures below we discuss the results of the computations. In particular, the term *α*_3_ is varied throughout the analysis since it captures a significant dynamical feature of this problem; the switching from on-pathway to off-pathway. Over all, we choose the following ranges for our parameters: ${10}^{-10}\le {\alpha }_{i},{\beta }_{i}\le {10}^{10}$ for *i* = 1, 2. Also, $1\le n\le 20$ and $1\le {B}_{1,e}\le 20$. We begin with the *base case* defined by the choice ${\alpha }_{1}={\beta }_{1}=0.1$, ${\alpha }_{2}={\beta }_{2}=100$, *n* = 4 and *B*_1,*e*_ = 1 and then study the sensitivity of the system and our results to each of the parameters.

### Ensemble Kinetic Simulations (EKS) of on- and off-pathways

#### Experimental observations and model assumptions

The detailed simulations and subsequent analysis are based on prior work where the *in-vitro* experiment using the non-esterified fatty acids of chain length C9 to C12 revealed a few crucial characteristics of A*β*42 aggregation^22^:

1. At *FA* concentration less than CMC range (*FA*_*n*_), only on-pathway aggregates were produced. Furthermore an increase in ThT intensity than the control experiment was detected.
2. At near CMC (*FA*_*pm*_), off-pathway fibrils of length 12–24 mers were noticed with very few fibrils being produced leading to the conclusion that the on-pathway was almost switched off.
3. At *FA* concentration higher than CMC (*FA*_*m*_), off-pathway oligomers of length 4–5 mers were observed while no fibrils were produced, i.e., the on-pathway was switched off completely.

Before establishing a proper modeling of the off-pathway, we wish to explore the formation of micelles first. Ideally, CMC is the concentration of the surfactants (here, *FA*s) in the solution from where the micelle formation starts. Therefore, if high concentration of surfactants are introduced into the solution (*FA*_*m*_), it directly forms micelles. At *FA*_*n*_ and *FA*_*pm*_ concentrations, ideally there should not be any micelles present in the solution whereas at *FA*_*m*_ range, almost all of the *FA*s should convert into micelles. As discussed earlier, at *FA*_*pm*_, the *FA*s loosely form pseudo-micelles that are six-seven times larger than regular micelles. It can hence be hypothesized that as the *FA* concentration is increased beyond the CMC, extra *FA* molecules bind to the pseudo-micelles, which may then undergo structural change to form more stable and compact structured micelles (i.e., *L*) that were considered in the ROM model.

Based on the experimental observations, we make the following considerations to formulate the underlying reaction mechanism.

1. At *FA*_*n*_ range, only on-pathway reactions take place, as in this zone no micelles are present in the solution. We assume that the presence of *FA*s have catalytic effects in altering the rate constants of the on-pathway with a factor of *K* resulting in a change in the final fibril concentration; the corresponding set of reactions are shown in Appendix-C under Supplementary Information.
2. At *FA*_*m*_ concentrations, both on-pathway and off-pathway occur simultaneously; however, as seen in the ROM model, the existence of micelles can switch the A*β* monomers more towards the off-pathway aggregates. We further assume that one micelle binds four A*β* monomers at once to form the off-pathway species ${A}_{4}^{^{\prime} }$; such ${A}_{4}^{^{\prime} }$ oligomers cannot aggregate any further. This is because our experimental observations at *FA*_*m*_ range point to the formation of ${A}_{4}^{^{\prime} }{\rm{s}}$ which do not aggregate to form fibrils.
3. Similarly at *FA*_*pm*_ range, both on- and off-pathway reactions occur simultaneously. The reaction mechanism for ${A}_{4}^{^{\prime} }$ oligomer formation is similar as in the previous step. However, in this case the oligomer ${A}_{4}^{^{\prime} }$ further aggregates to form 12–24 mers.

#### On-pathway reaction model

The on-pathway reaction model is motivated by our previous works in refs 9, 10, 47, 52, 56. Here, two different sets of reactions are considered. Firstly, the A*β* monomers form higher order aggregates (*A*_12_) through monomer addition and eventually form fibrils; the modeling abstraction considers *A*_12_ as fibrils, *F*. These reactions mainly depend on the monomer concentration and are slow being termed as pre-nucleation. Next, the higher order oligomers (*A*_12_) react with the monomers and other on-pathway aggregates (*A*_2_, …, *A*_11_) and get elongated. The rates of these reactions depend on both monomer and fibril concentration. The pre-nucleation stage is slower, whereas the elongation phase is rapid, thereby causing the sigmoidal growth of the fibril concentration over time. The nucleation number of on-pathway reactions is taken as 12 as reported in ref. 48; these reactions are shown in Appendix-B under Supplementary Information.

#### *FA*_*n*_ reaction model

At the *FA*_*n*_ range, we assume the reactions to be exactly similar to the on-pathway model; however the rate constants for each phase of the sigmoidal ThT growth curve were varied by a factor of *K*. The corresponding reaction model is shown in Appendix-C under Supplementary Information.

#### *FA*_*m*_ reaction model

The proposed off-pathway reaction set is quite different from the on-pathway model and motivated by the experimental observations. In this model, at *FA*_*m*_ range, the four A*β* monomers directly convert into the off-pathway oligomer (${A}_{4}^{^{\prime} }$) by reacting with micelles, *L*. These ${A}_{4}^{^{\prime} }{\rm{s}}$ can not able elongated any further. These reactions are shown in Appendix-E under Supplementary Information.

#### *FA*_*pm*_ reaction model

The modeling of off-pathway reactions at *FA*_*pm*_ zone is more challenging however; there are no existing models for off-pathway reactions at this zone. Furthermore, the behavior of aggregation, although sigmoidal, is much dissimilar than the on-pathway reaction in terms of the faster time-scale involved; the formation of 12–24 mers in this zone exhibit considerably less lag time and saturation time. We consider the following reactions at this range. In the first stage denoted as primary nucleation, the ${A}_{4}^{^{\prime} }$ form higher order oligomers (${A}_{12}^{^{\prime} }$) by monomer addition (i.e., adding *A*_1_). In the next stage, ${A}_{12}^{^{\prime} }$ further elongates with the intermediate oligomers (${A}_{4}^{^{\prime} }$–${A}_{11}^{^{\prime} }$) to form higher order oligomers (${F}_{1}^{^{\prime} }$); this stage is termed as the elongation stage. Note that a ${F}_{1}^{^{\prime} }$ is a modeling abstraction and is of variable length; it’s length ranges from 16 mers to 23 mers (considering the addition of ${A}_{4}^{^{\prime} },\mathrm{...},{A}_{11}^{^{\prime} }$ each to *A*_12_). This results in a 6-fold increase in the oligomer size hosted by the pseudo-micelles as compared to the micelles (which can only host upto ${A}_{4}^{^{\prime} }$, whereas pseudo-micelles can host 23 mers) and is consistent with the experimental observations on the diameters of pseudo-micelles and micelles. We next consider such ${F}_{1}^{^{\prime} }$ oligomers to laterally associate and create bigger oligomers (${F}_{4}^{^{\prime} }$); we term this stage as lateral association. As experimentally validated in Fig. 6, there is approximately a four-fold increase in oligomer size towards the beginning of the *FA*_*pm*_ dynamics after which the oligomer size goes down to that of ${A}_{12}^{^{\prime} }$ and ${F}_{1}^{^{\prime} }$; hence it is highly likely that the laterally associated ${F}_{4}^{^{\prime} }$ undergoes a secondary fragmentation into the lower order oligomer ${F}_{1}^{^{\prime\prime} }$.

It is noteworthy that ${F}_{1}^{^{\prime\prime} }$ is a structurally different species than ${F}_{1}^{^{\prime} }$; the latter can laterally associate while the former cannot. This assumption was necessary to fit with the experimentally observed ThT kinetics data. Considering the secondary fragmentation to produce ${F}_{1}^{^{\prime} }$ instead of ${F}_{1}^{^{\prime\prime} }$ could not correlate the simulation plots with the experimentally observed ThT dynamics. The set of near-CMC reactions are shown in Appendix-D under Supplementary Information.

Thus, the model at the near-CMC range deals with several rate constants and parameters. There are four rate constants for on-pathway reactions: (i) forward and backward rate constants of pre-nucleation reaction (*k*_*nuon*_, *k*_*nuon*__) from the on-pathway; (ii) forward and backward rate constants of elongation reactions (*k*_*fbon*_, *k*_*fbon*__) from the on-pathway; (iii) forward and backward rate constants of pre-nucleation in the off-pathway (*k*_*con*_ and *k*_*con*__); (iv) forward and backward rate constants of nucleation reactions (*k*_*nouff*_, *k*_*nouff*__); (v) forward and backward rate constants of elongation reactions (*k*_*fboff*_, *k*_*fboff_*_) (vi) forward and backward rate constants of lateral association reactions (*k*_*eloff*_, *k*_*eloff_*_); (vii) forward and backward rate constants of secondary fragmentation (*k*_*fagoff*_, *k*_*fagoff_*_); (viii) the fatty acid effect on the on-pathway rate constant parameter, *K*; and finally (ix) the pseudo-micelle concentration: *p*. Note that, the estimation of the pseudo-micelle concentration from the CMC values of fatty acids poses a different problem and needs controlled experimentation; however, it is biophysically not possible to exactly measure the pseudo-micelle concentration. We could only measure the diameter of pseudo-micelles. Hence we considered *p* as a free parameter along with the other rate constants to estimate the pseudo-micelle concentration.

#### Parameter estimation

It is difficult to estimate the proper rate constant values of all these parameters at once. Hence, we follow our divide and conquer strategy from ref. 10 to determine all the rate constants step by step. First, we matched the experimental data of the control experiment with the simulation considering the on-pathway reaction setup only and estimated the four rate constants involved in the on-pathway reactions. Subsequently, we used these values in the combined on-off-pathway model to estimate all the additional rate constants. More precisely, our simulation involves the steps below:

1. First, determine the rate constants for the on-pathway reactions from control experiment data.
2. Estimate the parameter *K* for the *FA*_*n*_ experimental data by using the estimated on-pathway rate constants.
3. Estimate the rate constants of off-pathway aggregation with *FA*_*pm*_ experimental data utilizing the estimated on-pathway rate constants above.
4. Validate the formation of off-pathway oligomers (${A}_{4}^{^{\prime} }$) in *FA*_*m*_ concentration using all the estimated rate constants.

We first calculated the reaction flux for all reactions at a particular stage; then using these reaction fluxes, the differential equations for the rate of change of concentration is formulated for each oligomer. Next, these differential equations were solved using MATLAB’s ode solver and the *R* ^2^ value between the simulated curve and experimental data was calculated for the different rate constant combinations. We solved these differential equations for various rate constant combinations with each rate constant ranging from 10^−5^ to 10^5^ and the pseudo-micelle concentration *p* from 1 to 100 *μM* with multiples of 5. After that, these rate constants were manually fine-tuned to better match the experimental data and obtain better rate constant estimates. The best-fitted simulation parameters are taken as estimated rate constants of these reactions; each of these parameters are reported in the Appendices under the corresponding reaction models. Moreover, all the rate constants estimated here by fitting with experimental ThT intensity plots required a mapping of the cumulative effects of the concentration of higher order ThT positive oligomers to the ThT values; such mapping is explained in Appendix-F in the Supplementary Information.

## Electronic supplementary material

Supplementary Information
