# Supplementary material for: Fatty Acid Concentration and Phase Transitions Modulate Aβ Aggregation Pathways
Source: Sci Rep. 2017 Sep 4;7:10370. doi: 10.1038/s41598-017-09794-x (PMC5583381; doi:10.1038/s41598-017-09794-x)
Supplement: Supplementary file 1 — Supplementary Information [file 41598_2017_9794_MOESM1_ESM.pdf]

Fatty Acid Concentration and Phase Transitions Modulate Aβ Aggregation Pathways

Pratip Rana [1], Dexter N. Dean [2], Edward D. Steen [3],
Ashwin Vaidya [3], Vijayaraghavan Rangachari [2] and Preetam Ghosh [1*]
[1] *Department of Computer Science, Virginia Commonwealth University, Richmond, VA 23284*
[2] *Department of Chemistry & Biochemistry, University of Southern Mississippi, Hattiesburg, MS 39406*
[3] *Department of Mathematical Science, Montclair State University, Montclair, NJ 07043*
[*] *Corresponding email: pghosh@vcu.edu*

Appendix A: ROM switching behavior

↓ Significant deviation from base case Fig. 3 in main manuscript	α_1	α_2	β_1	β_2
10^{-10}	Single switch; OFP becomes more stable for $\alpha_3 > 1$	No switching; ONP more stable	Single switch; OFP more stable ; for $\alpha_3 > 1$	No switch ONP more stable
10^{-2}	Base case; Two switching points at $\alpha_3 = 1$, $\alpha_3 = 1.87$		Base case; Two switching points at $\alpha_3 = 1$, $\alpha_3 = 1.87$	
10^{0}			Both switching points move to the left	
10^{2}		Base case; Two switching points $\alpha_3 = 1$, $\alpha_3 = 1.87$		Base case; Two switching points $\alpha_3 = 1$, $\alpha_3 = 1.87$
10^{4}	First critical α_3 vanishes; second critical $\alpha_3 \to 1.93$	No switching; ONP more stable	Only one switch; ONP more stable for $\alpha_3 > 0.6$	Three switching points; $\alpha_3 \approx 0.3$, $\alpha_3 \approx 1.2$, $\alpha_3 \approx 1.4$; ONP stable for $\alpha_3 \approx 1.4$
10^{6}	ONP always more stable		No switch	Single switch for small α_3
10^{10}			Single switch; OFP more stable for small α_3	

Table 1: The table shows significant changes in the switching behavior of the system with changes to the values of the parameters. ONP refers to on-pathway and OFP to off-pathway.

Parameter	Predicted Value
k_{nuon}	$1 * 10^{-2} \mu M^{-1} h^{-1}$
k_{nuon_-}	$1 * 10^{-4} h^{-1}$
k_{fbon}	$4 * 10^{3} \mu M^{-1} h^{-1}$
k_{fbon_-}	$6 * 10^{0} h^{-1}$

Table 2: Predicted reaction parameters of on-pathway from control experiment

Appendix B: EKS: On-pathway model

Following set of reactions were considered for the combined on-off pathway simulation model.

Reactions considered in the nucleation stage involve monomer additions and are as follows:

$$A_1 + A_1 \xleftrightarrow[k_{nuon_-}]{k_{nuon}} A_2 \tag{1}$$

$$\ldots\ldots \tag{2}$$

$$A_{11} + A_1 \xleftrightarrow[k_{nuon_-}]{k_{nuon}} A_{12} \tag{3}$$

In the elongation stage, all on-pathway A_{12}'s abstracted as fibrils, F, elongate using the lower molecular weight oligomers. To simplify the model, we have grouped all the higher order oligomers of size A_{12} and above as a single species termed the fibril, F. The reactions for the elongation stage are as follows:

$$A_{12} + A_1 \xleftrightarrow[(k_{elon_-})]{k_{elon}} F \tag{4}$$

$$A_{12} + A_i \xleftrightarrow[(k_{elon_-})]{k_{elon}} F; i = 1, ..., 11 \tag{5}$$

$$A_{12} + A_{11} \xleftrightarrow[(k_{elon_-})]{k_{elon}} F \tag{6}$$

Flux of the reactions at the nucleation stage are given by

$$H_1 = k_{nuon}[A_1][A_1] - k_{nuon_-}[A_2] \tag{7}$$

$$\cdots \tag{8}$$

$$H_i = k_{nuon}[A_i][A_1] - k_{nuon_-}[A_{i+1}]; \forall i \in \{1, 2, \cdots, 11\} \tag{9}$$

Flux of the reactions at the elongation stage are as follows:

$$I_1 = k_{fbon}[A_1][F] - k_{fbon_-}[F] \tag{10}$$

$$\cdots \tag{11}$$

$$I_i = k_{fbon}[A_i][F] - k_{fbon_-}[F]; \forall i \in \{1, 2, \cdots, 11\} \tag{12}$$

The rate of change of monomer concentration can hence be written as

$$\frac{\mathrm{d}A_1}{\mathrm{d}t} = -I_1 - \sum_{i=1}^{11} H_i \tag{13}$$

The differential equations explaining the rate of change of each species concentration produced in the nucleation stage ($A_2 - A_{11}$) is given by

$$\frac{\mathrm{d}A_{i-1}}{\mathrm{d}t} = -H_i + H_{(i-1)} - I_i; \forall i \in \{2, \cdots, 11\} \tag{14}$$

Finally, the differential equation for the rate of change in concentration of F is

$$\frac{\mathrm{d}F}{\mathrm{d}t} = H_{11} \tag{15}$$

The estimated rate constants from this stage are shown in Table 2.

FA chain length	Predicted Value of K	R^2
$C9$	0.7	75.2
$C10$	0.6	94.7
$C11$	1.0	89.4
$C12$	0.85	90.3

Table 3: Predicted value of parameter K for different fatty acid chain lengths

Appendix C: EKS: Below-CMC (FA_n) model

Reactions at below-CMC are also quite similar with that of control (i.e., the on-pathway). At below-CMC zone, only the on-pathway reactions are considered. But here, in the elongation stage the forward rate constant was modified by a factor of K due to the presence of fatty acids. Note that in our simulations we considered the fact that each of the rate constants from the on-pathway could be altered by the off-pathway; however, only a change in the forward elongation rate constant gave us the best match with the experimental ThT plots.

The reactions from the elongation stage are as follows:

$$A_{12} + A_1 \xrightleftharpoons[(k_{elon_-})]{K*k_{elon}} F \tag{16}$$

$$A_{12} + A_i \xrightleftharpoons[k_{elon_-}]{K*k_{elon}} F; n = 1, ..., 10 \tag{17}$$

$$A_{12} + A_{11} \xrightleftharpoons[k_{elon_-}]{K*k_{elon}} F \tag{18}$$

Flux of the reactions from the elongation stage are given by

$$I_1^* = K * k_{fbon}[A_1][F] - k_{fbon_-}[F] \tag{19}$$

$$\cdots \tag{20}$$

$$I_i^* = K * k_{fbon}[A_i][F] - k_{fbon_-}[F]; \forall i \in \{1, 2, \cdots, 11\} \tag{21}$$

The rate of change of concentration of Aβ monomers can hence be written as

$$\frac{\mathrm{d}A_1}{\mathrm{d}t} = -I_1^* - \sum_{i=1}^{11} H_i \tag{22}$$

The differential equations for each species produced in the primary nucleation stage ($A_2 - A_{11}$) are as follows

$$\frac{\mathrm{d}A_{i-1}}{\mathrm{d}t} = -H_i + H_{(i-1)} - I_i^*; \forall i \in \{2, \cdots, 11\} \tag{23}$$

The derivation of concentration change rates of F remains the same as in the on-pathway.

The estimated values of K and corresponding R^2 with experimental fits from this stage are shown in Table 3. Additionally, Fig 1 shows the plots for the normalized versions of the experimental data and simulated curves.

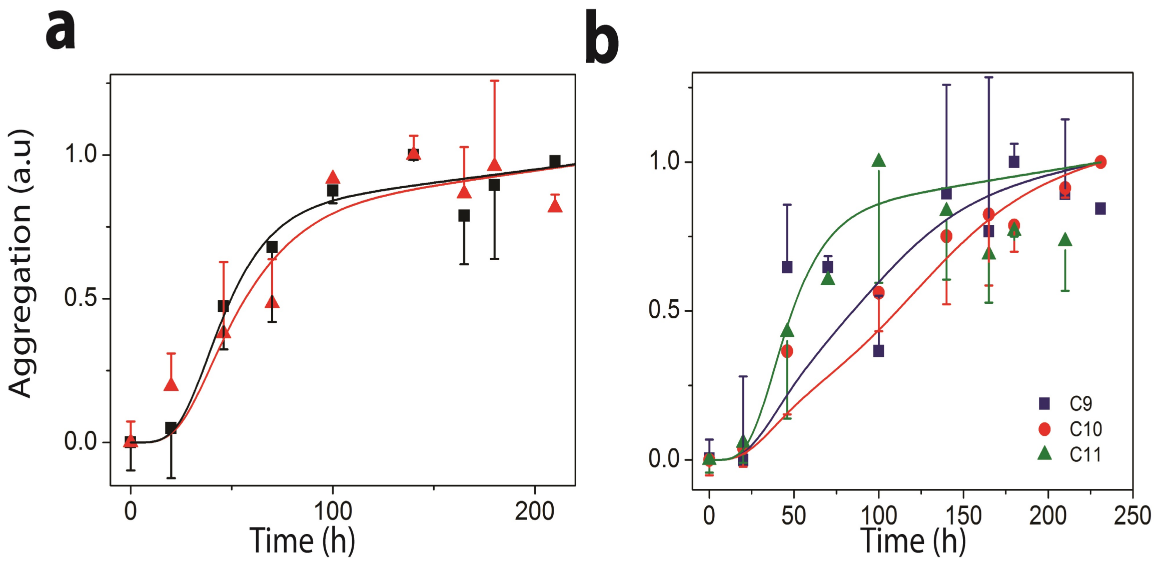

Figure 1: Normalized experimental and simulated data at control and below CMC concentration. (a) For C12 FAs; Black squares: Experimental ThT intensity for on-pathway; Black line: simulated ThT intensity for on-pathway; Red triangles: Experimental ThT intensity for below-CMC set-up; Red line: simulated ThT intensity for below-CMC set-up; (b) Below-CMC experimental ThT intensity and simulated intensities for C9-C11.

Appendix D: EKS: Near-CMC (FA_{pm}) model

At the near-CMC zone, both on-pathway and off-pathway reactions occur simultaneously. The on-pathway reactions are shown in Appendix B. Amongst the off-pathway reactions, the first stage is pre-nucleation where following reaction was considered.

$$4A_1 + L \underset{k_{con}}{\overset{k_{con}}{\longleftrightarrow}} A'_4 \tag{24}$$

The next stage is termed primary-nucleation and the corresponding reactions are

$$A'_4 + A_1 \underset{k_{nuoff_-}}{\overset{k_{nuoff}}{\longleftrightarrow}} A'_5 \tag{25}$$

$$...... \tag{26}$$

$$A'_{11} + A_1 \underset{k_{nuoff_-}}{\overset{k_{nuoff}}{\longleftrightarrow}} A'_{12} \tag{27}$$

Finally, in the off-pathway elongation stage, all off-pathway fibrils elongate using the low molecular weight oligomers. Due to these reactions the fibril concentration saturates after some time period. Now consideration of the exact reactions in this stage is complicated as we need to consider fibrils of different lengths. To simplify the model, we group all fibrils of length $12 - 23$mers as a single species: F'_1. Hence, the reactions for the elongation stage are:

$$A'_{12} + A'_4 \underset{k_{eloff_-}}{\overset{k_{eloff}}{\longleftrightarrow}} F'_1 \tag{28}$$

$$A'_{12} + A'_n \underset{k_{eloff_-}}{\overset{k_{eloff}}{\longleftrightarrow}} F'_1; i = 5, ..., 10 \tag{29}$$

$$A'_{12} + A'_{11} \underset{k_{eloff_-}}{\overset{k_{eloff}}{\longleftrightarrow}} F'_1 \tag{30}$$

In the lateral association stage, the off-pathway fibrils associate with themselves and form higher molecular weight structures. The reactions involved in this stage are as follows.

$$F'_1 + F'_1 \underset{k_{fboff_-}}{\overset{k_{fboff}}{\longleftrightarrow}} F'_2 \tag{31}$$

$$...... \tag{32}$$

$$F'_3 + F'_1 \underset{k_{fboff_-}}{\overset{k_{fboff}}{\longleftrightarrow}} F'_4 \tag{33}$$

Finally, we consider a secondary fragmentation stage in the off-pathway as follows:

$$F'_4 \underset{k_{fagoff_-}}{\overset{k_{fagoff}}{\longleftrightarrow}} 4F''_1 \tag{34}$$

Reaction flux for the pre-nucleation stage can be computed as:

$$G'_1 = k_{con}[A_1]^4[L] - k_{con_-}[A'_4] \tag{35}$$

Flux of the reactions in the primary nucleation stage are

$$H'_1 = k_{nuoff}[A'_4][A_1] - k_{nuoff_-}[A'_5] \tag{36}$$

$$\cdots \tag{37}$$

$$H'_i = k_{nuoff}[A'_{4+i-1}][A_1] - k_{nuoff_-}[A'_{4+i}]; \forall i \in \{1, 2, \cdots, 8\} \tag{38}$$

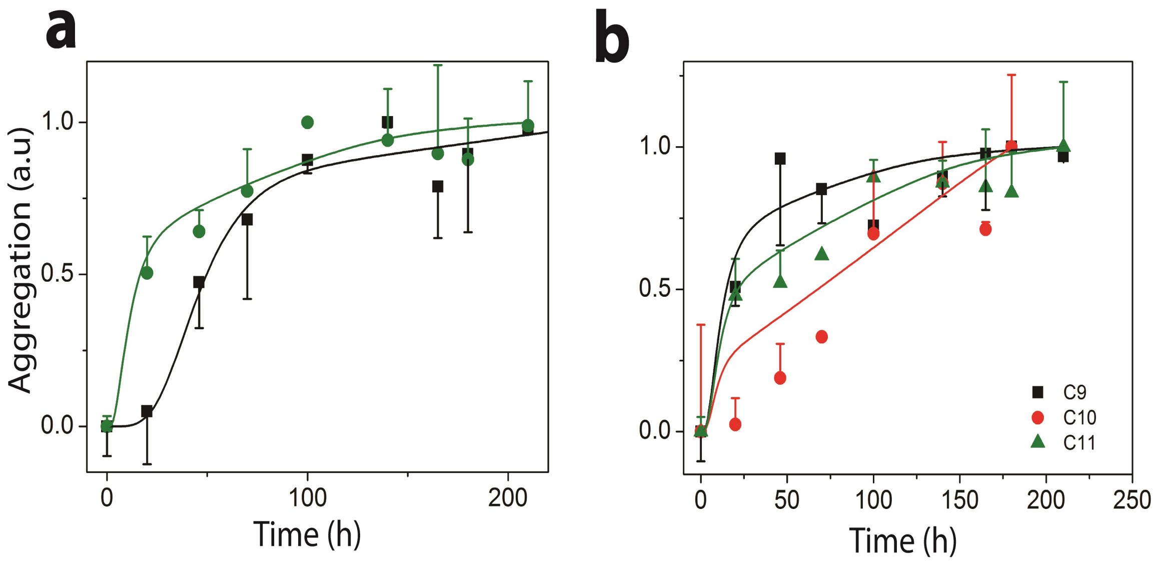

Figure 2: Normalized experimental and simulated data at near CMC concentration: (a) Green circles: Experimental ThT intensity for near-CMC set-up; Green line: simulated ThT intensity for near-CMC set-up; Black squares: Experimental ThT intensity for on-pathway; Black line: simulated ThT intensity for on-pathway; (b) Near-CMC experimental ThT intensity and simulated intensities for C9-C11.

Parameter	Predicted Value
k_{con}	$1 * 10^{-4} \mu M^{-3} h^{-1}$
k_{con_-}	$0h^{-1}$
k_{nuoff}	$8.5 * 10^{-2} \mu M^{-1} h^{-1}$
k_{nuoff_-}	$1 * 10^{-2} h^{-1}$
k_{fboff}	$1 * 10^{4} \mu M^{-1} h^{-1}$
k_{fboff_-}	$2 * 10^{-1} h^{-1}$
k_{laoff}	$1 * 10^{-2} \mu M^{-1} h^{-1}$
k_{laoff_-}	$5 * 10^{-3} h^{-1}$
k_{fagoff}	$5 * 10^{3} h^{-1}$
k_{fagoff_-}	$6 * 10^{-7} \mu M^{-3} h^{-1}$

Table 4: Additional predicted reaction parameters of off-pathway at the FA_{pm} zone

Flux of the reactions in the elongation stage are

$$I'_1 = k_{fboff}[A'_4][A'_{12}] - k_{fboff\_}[F'_1] \tag{39}$$
$$\cdots \tag{40}$$
$$I'_i = k_{fboff}[A'_{4+i-1}][A'_{12}] - k_{fboff\_}[F'_1]; \forall i \in \{1, 2, \cdots, 8\} \tag{41}$$

Similarly fluxes of the lateral association stage are given by:

$$P'_1 = k_{laoff}[F'_1][F'_1] - k_{laoff\_}[F'_2] \tag{42}$$
$$\cdots \tag{43}$$
$$P'_i = k_{laoff}[F'_i][F'_1] - k_{laoff\_}[F'_{i+1}]; \forall i \in \{1, 2, \cdots, 3\} \tag{44}$$

Finally, the reaction flux of the secondary fragmentation stage is given by

$$R'_1 = k_{fagoff}[F'_4] - k_{fagoff\_}[F''_1]^4 \tag{45}$$

Next, the species specific differential equations of reaction rates were formulated. The monomer concentration change rate can be written as

$$\frac{\mathrm{d}A_1}{\mathrm{d}t} = -4G'_1 - \sum_{i=1}^{8} H'_i - I_1 - \sum_{i=1}^{11} H_i \tag{46}$$

Differential equation formulation of $A_2 - A_{11}$ and F is the same as Appendix B. Differential equation for A'_4 can be written as

$$\frac{\mathrm{d}A'_4}{\mathrm{d}t} = G'_1 - H'_1 - I'_1 \tag{47}$$

The differential equation of each species produced in the primary nucleation stage ($A'_5 - A'_{11}$) is given by

$$\frac{\mathrm{d}A'_{4+i-1}}{\mathrm{d}t} = -H'_i + H'_{(i-1)} - I'_i; \forall i \in \{2, \cdots, 8\} \tag{48}$$

The derivation of concentration of A'_{12} is given by

$$\frac{\mathrm{d}F'_1}{\mathrm{d}t} = H'_8 - P'_1 - \sum_{i=1}^{3} P'_i \tag{49}$$

The derivative of the concentration for each oligomer produced through the lateral association stage can be written as

$$\frac{\mathrm{d}F'_i}{\mathrm{d}t} = P'_{i-1} - P'_i; \forall i \in \{2, \cdots, 3\} \tag{50}$$

Finally, the time derivative of the concentration of F'_4 can be written as

$$\frac{\mathrm{d}F'_4}{\mathrm{d}t} = P'_3 - R'_1 \tag{51}$$

And the derivative of concentration of F''_{12} will be

$$\frac{\mathrm{d}F''_1}{\mathrm{d}t} = 4R'_1 \tag{52}$$

The estimated rate constants from this stage are shown in Table 4. Additionally, Fig 2 shows the plots for the normalized versions of the experimental data and simulated curves.

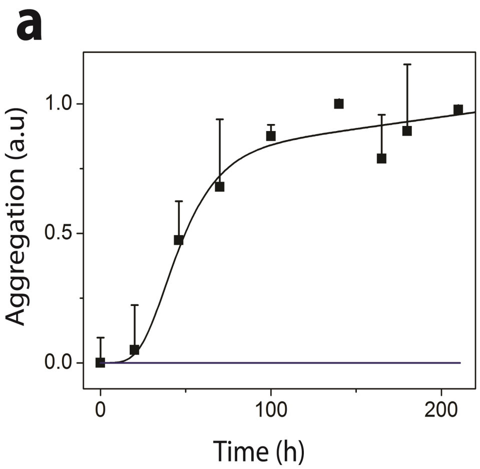

Figure 3: Normalized experimental (points) and simulated (lines) ThT intensities at above-CMC concentration; Black: on-pathway; Blue line: simulated ThT intensity at above-CMC set-up.

Appendix E: EKS: Above-CMC (FA_m) model

In the above CMC zone, on-pathway reactions and only the pre-nucleation stage reactions of the off-pathway occur simultaneously. The reactions and corresponding reaction flux formulation of on-pathway and off-pathway pre-nucleation stage is similar to that shown in Appendix D.

The monomer concentration change rate can be written as

$$\frac{\mathrm{d}A_1}{\mathrm{d}t} = -4G'_1 - I_1 - \sum_{i=1}^{11} H_i \tag{53}$$

Differential equation for A'_4 can be written as

$$\frac{\mathrm{d}A'_4}{\mathrm{d}t} = G'_1 \tag{54}$$

Differential equations of other on-pathway oligomers $A_2 - A_{11}$ and F will remain the same .

Fig 3 shows the normalized versions of the experimental data and simulated curves from the on-pathway and the ThT intensities from the SA_m phase; the latter is obviously zero as the largest oligomers from this phase, A'_4 are not considered ThT-positive.

Appendix F: Comparing simulated aggregation curves to ThT fluorescence intensities

The ThT fluorescence intensity plots from experiments essentially show the cumulative effect of all ThT positive oligomers of a certain size (and beyond). As the reactions considered in the above models primarily involve elongation through oligomer addition, they are accompanied by an increase in ThT fluorescence due to the generation of new ThT binding sites. Hence, from the simulation, one has to plot the cumulative effects from all the ThT-positive oligomers that can be mapped directly to the experimental estimates. In order to do this, we compute the following expression at each value of the simulation time:

$$For\ on-pathway\ and\ SA_n\ phases : [F] \times b_1; \tag{55}$$

$$For\ SA_{pm}\ phase : (\sum_{i=1}^{4}[F_i'] \times i + [F_1''] + [F]) \times b_2; \tag{56}$$

where $[F]$ denotes the concentration of F that designate the largest species for both the on-pathway and SA_n phases and are considered ThT-positive. This formulation is exactly similar to several other works which perform the abstraction of considering all larger oligomers beyond nucleation to have a similar effect on the ThT binding sites, most notably [1, 3, 4, 6]. Also, b_1 is simply a scaling constant to map the combined effect of the higher order oligomers onto the ThT intensity values; it is another free parameter in the model.

Similarly, for the SA_{pm} phase, $[F_i']$ and $[F_1'']$ denote the concentration of F_i' and F_1'' respectively; these were the off-pathway ThT positive species considered in the model apart from the simultaneously occurring on-pathway species. Also, b_2 is a scaling constant as before. Notably, here the size of the larger oligomers, i.e., $i, i \in 1, ..., 4$, is considered to have an increasing effect on the ThT intensities as shown in our previous works and existing literature [2, 5, 6].

Note that for the on-pathway (and the SA_n phase), F refers to the nucleated species and possibly undergoes a structural change to become ThT positive as considered by other models. Here, although the actual size of the fibrils are not considered explicitly due to the reaction abstraction, the concentration of the post-nucleation phase oligomers can approximately capture the effects on the ThT intensities. Here, the ThT binding sites are only incremented by elongation with pre-nucleation oligomers which are small ($A_1 - A_{11}$), and hence the actual size of F will not affect the ThT estimates significantly. Since the experimental ThT curves start from zero, it is expected that the pre-nucleation species that are instantaneously formed, do not affect the ThT intensities. For the SA_{pm} phase, similarly, it is expected that the oligomers undergo a structural change after the elongation stage; hence corresponding oligomeric species in the lateral association and secondary fragmentation phases are considered ThT positive.

References

[1] Ghosh, P., Datta B. & Rangachari V. Computational predictions for the lag-times and nucleation mass involved in Aβ42 peptide aggregation. *Proceedings of the International Conference on Bioinformatics Models, Methods and Algorithms (BIOINFORMATCIS)*, 312-316 (2012).

[2] Ghosh, P., Kumar, A., Datta, B. & Rangachari, V. Dynamics of protofibril elongation and association involved in Aβ42 peptide aggregation in Alzheimer's disease. *BMC Bioinformatics* **11** Suppl 6, S24 (2010).

[3] Ghosh, P., Vaidya, A., Kumar, A. & Rangachari, V. Determination of Critical Nucleation Number for a Single Nucleation Amyloid-β Aggregation Model. *J. Mathematical Biosciences* **273**, 70-79 (2016).

[4] Lee, C-C., Nayak, A., Sethuraman, A., Belfort, G. & McRae, G. J. A three-stage kinetic model of amyloid fibrillation. *Biophysical Journal* **92**, 3448-3458 (2007).

[5] Dean, D. N. et al. Strain-specific Fibril Propagation by an Abeta Dodecamer. *Scientific reports* **7**, 40787, (2017).

[6] Morris, A. M., Watzky, M. A. & Finke, R. G. Protein aggregation kinetics, mechanism, and curve-fitting: a review of the literature. *Biochim Biophys Acta* **1794**, pp. 375-397 (2009).

[7] Ghosh, P., Ghosh, S., Basu, K., Das, S.K. & Daefler, S. An analytical model to estimate the time taken for cytoplasmic reactions for stochastic simulation of complex biological systems. *Granular Computing, 2006 IEEE International Conference on*, 79-84 (2006).
